# Disruption of spike protein N-glycosylation induces its endoplasmic reticulum retention and attenuates SARS-CoV-2 infectivity

**DOI:** 10.1128/jvi.00270-26

**Published:** 2026-03-30

**Authors:** Weili Kong, Jiali Zhang, Yingying Song, Jingjing Song, Yuebo Xu, Xinmu Xu, Haoyu Ma, Li Chen, Cong Zeng

**Affiliations:** 1 Key Laboratory of Medical Molecular Virology (MOE/NHC/CAMS), Shanghai Frontiers Science Center of Pathogenic Microorganisms and Infection, Shanghai Institute of Infectious Disease and Biosecurity, School of Basic Medical Sciences, Shanghai Medical College, Fudan University 58305, Shanghai, China; University of Minnesota Twin Cities, Minneapolis, Minnesota, USA

**Keywords:** spike N-glycosylation, protein trafficking, SARS-CoV-2, MOGS

## Abstract

The spike (S) protein of SARS-CoV-2 is extensively glycosylated, with N-glycosylation sites remaining highly conserved during viral evolution. While inhibiting N-glycosylation has been shown to significantly suppress SARS-CoV-2 infection, the underlying molecular mechanisms remain incompletely characterized. Here, we identify that two N-glycosylation sites, N61 and N343, are critical for spike maturation. We demonstrate that asparagine-to-aspartic acid substitutions (N to D) at these sites lead to endoplasmic reticulum (ER) retention of the S protein, with consequent abrogation of S1/S2 cleavage and near-complete elimination of viral infectivity. IP-MS analysis further reveals that the COPI complex, which facilitates retrograde Golgi-to-ER transport, is a key participant in this ER retention process. Additionally, inhibition of COPI effectively restores the plasma membrane localization of N61D- and N343D-mutated S proteins and enhanced viral infectivity. More importantly, a specific inhibitor has been developed that effectively blocks the ER-to-Golgi trafficking of the S protein, thereby broadly abolishing viral infectivity across SARS-CoV-2 variants. Overall, our study reveals the unique roles of N-glycosylation in the regulation of S protein maturation, providing a potential mechanistic target for antiviral drug development.

**IMPORTANCE:**

N-glycosylation of the spike protein is critical for SARS-CoV-2. While most studies have focused on the effects on spike-ACE2 binding and neutralizing antibody recognition, few studies have reported how N-glycosylation regulates S protein maturation, with the underlying molecular mechanisms remaining poorly understood. Here, we demonstrate that N-glycosylation at N61/ N343 contributes to spike ER-to-Golgi trafficking. Specifically, defects in S protein’s N-glycosylation (including mutations at N61 or N343, N-glycosylation inhibitors treatment, and MOGS depletion) result in ER retention through COPI-mediated retrograde Golgi-to-ER transport, and thus, the S proteins are not effectively cleaved by furin in the Golgi. This impairment of S protein maturation leads to a significant reduction in viral infectivity, which highlights the key role of N-glycosylation at residues N61 and N343 in SARS-CoV-2 life cycle. Overall, our findings uncover the molecular mechanism by which N-glycosylation controls SARS-CoV-2 spike intracellular trafficking, offering novel insights for anti-SARS-CoV-2 strategies.

## INTRODUCTION

Glycosylation is an important post-translational or co-translational modification of proteins in eukaryotes. N-linked glycosylation, a prevalent glycosylation form, is critical for protein folding, structural stability, and diverse cellular functions (1, 2). The synthesis and transfer of N-glycans primarily occur in the endoplasmic reticulum (ER), with subsequent modifications taking place in both the ER and the Golgi apparatus. This process requires the involvement of multiple glycosyltransferases and glycosidases, under whose action multiple monosaccharide molecules are assembled in a non-templated manner and linked to specific amino acid residues of proteins via glycosidic bonds to form glycans (3). Deleterious variants in any of the glycosyltransferases or glycosidases can result in improper glycosylation of proteins or lipids, thereby causing congenital disorders of glycosylation (CDGs) (2, 4–7). MOGS (mannosyl-oligosaccharide glucosidase), also known as α-glucosidase I, is the first enzyme involved in the processing of N-linked glycans after their transfer from donor to proteins. MOGS is in the ER lumen and specifically cleaves terminal α-1,2-linked glucose residues from Glc3Man9GlcNAc2 (4, 8). Biallelic pathogenic variants in *MOGS* lead to a functional deficiency of this key enzyme, resulting in MOGS-CDG (9–11). Interestingly, MOGS-CDG patients exhibit reduced susceptibility to certain enveloped viruses (12). However, the underlying mechanism remains unclear.

Viruses, as small and structurally simple parasitic organisms, contain numerous glycosylated proteins on their surfaces, such as the envelope protein (gp120) of human immunodeficiency virus (HIV), the spike protein (S) of SARS-CoV-2, the hemagglutinin (HA) of the H1N1 influenza virus, the glycoprotein (GP) of Ebola virus (EBOV), the envelope glycoproteins (E1/E2) of hepatitis C virus (HCV), and the surface glycoproteins of hepatitis B virus (HBV) (13–23). These viral proteins and their surface glycans are closely related to viral replication, packaging, and transmission (24). Additionally, the glycans on the surface of viral proteins facilitate immune evasion through a “glycan shield” (25, 26). Furthermore, abnormal glycosylation of viral glycoproteins in herpes simplex virus type 2 (HSV-2), HBV, HIV, and other viruses impairs viral protein expression and infectious particle production (27–30). On the other hand, host cell glycans are critical for viral entry during infection. For example, charged sialic acid molecules on host cell surfaces enhance viral recognition of influenza virus and rotavirus infection (31, 32). Sialic acid glycoproteins have also been found to synergize with TMPRSS2, facilitating the entry of HCoV-HKU1 (33). These studies highlight the vital role of protein glycosylation in the viral life cycle, involving both viral proteins and host factors during key processes.

Severe acute respiratory syndrome coronavirus 2 (SARS-CoV-2) is an enveloped, positive-sense, single-stranded RNA virus of the genus *Betacoronavirus* (34, 35). During SARS-CoV-2 replication, viral protein synthesis and subsequent virion assembly strictly depend on the protein trafficking pathways of host cells. After the replication and transcription of viral genomic RNA in double-membrane vesicles (DMVs), structural proteins (S, M, E, N) are synthesized and subsequently transported to the ER-Golgi intermediate compartment (ERGIC) for virion assembly. Maturation processes, including glycosylation and furin-mediated S1/S2 cleavage, subsequently occur in the Golgi apparatus (36–39). Following assembly and maturation, SARS-CoV-2 virions are released from host cells by budding from the ERGIC or Golgi apparatus. The S protein of released progeny virions binds to the host receptor ACE2, mediating membrane fusion and thereby facilitating new infections (40, 41). Similar to other coronaviruses (37, 42), a dibasic retrieval signal (KxHxx) in the cytoplasmic tail of the SARS-CoV-2 S protein allows its recycling *via* binding to COPI (coat protein complex I) (43). This recycling of the S protein may increase its chance of interacting with the M protein, which resides at the ERGIC, thereby promoting S protein accumulation at the virion budding site (44). Notably, 22 N-linked glycosylation sites and 17 O-linked glycosylation sites have been identified in the SARS-CoV-2 S protein (22). Recent studies have highlighted the critical role of S protein N-glycosylation in modulating viral infectivity, spike-mediated receptor binding, and antigenicity (45, 46). Nevertheless, the mechanism by which N-glycosylation regulates S protein maturation during endomembrane trafficking remains a fundamental knowledge gap in SARS-CoV-2 research.

In this work, we elucidate the critical regulatory role of N-glycosylation in SARS-CoV-2 S protein maturation and viral infectivity. Mechanistic studies reveal that the proper N-glycosylation at the N61 and N343 sites contributes to S protein trafficking. Specifically, mutations at these sites lead to S protein retention in the ER, thereby preventing S1/S2 cleavage, which normally occurs in the Golgi apparatus, and thus blocking the formation of infectious viral particles. This ER retention of the mutant S protein is mechanistically linked to the retrograde transport pathway (Golgi to ER) mediated by the COPI complex. Furthermore, we have designed a polypeptide inhibitor (TAT-SIM) that targets the COPII-mediated anterograde transport pathway (ER to Golgi), which specifically induces ER retention of the S protein to mimic N-glycosylation defects, inhibiting viral infectivity by preventing S protein maturation. Owing to its conserved targeting sequence, TAT-SIM exhibits broad-spectrum antiviral activity and high specificity against all SARS-CoV-2 variants. Additionally, we demonstrate that MOGS deficiency disrupts the normal N-glycosylation of the S protein, leading to maturation defects and thereby revealing the potential molecular mechanism of viral resistance in MOGS-CDG patients. Collectively, this research not only reveals a pioneering mechanism by which N-glycosylation of viral proteins regulates infectivity but also provides a promising target for the development of broad-spectrum drugs against SARS-CoV-2 variants.

## RESULTS

### Suppression of host N-glycosylation diminishes the infectivity of SARS-CoV-2

**Fig 1:**
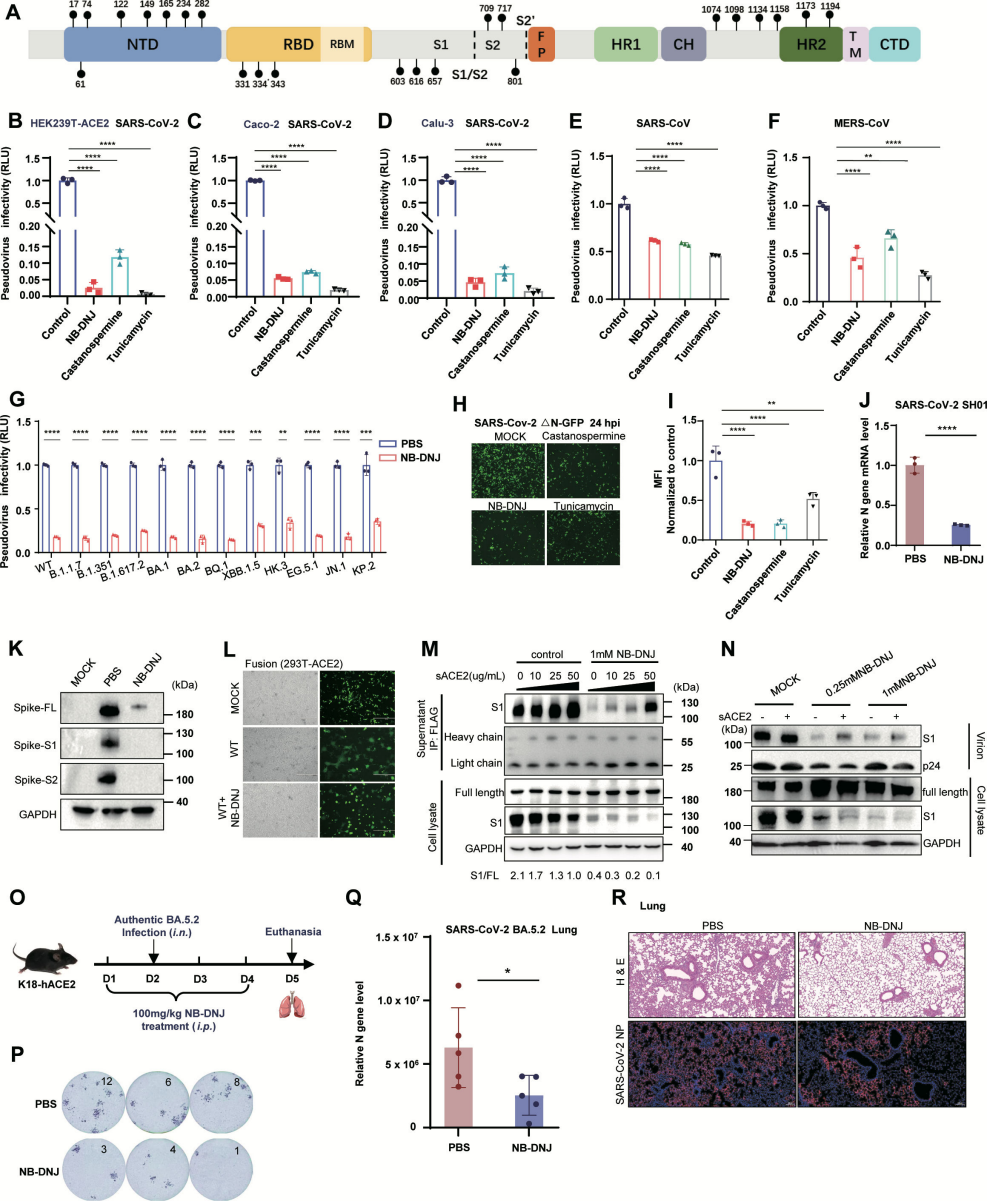
Suppression of host N-glycosylation diminishes the infectivity of SARS-CoV-2. (**A**) Schematic diagram of the SARS-CoV-2 spike (S) glycoprotein. N-linked glycosylation sites are depicted as branched structures. The following protein domains are labeled: the N-terminal domain (NTD), receptor binding domain (RBD), fusion peptide (FP), heptad repeat 1 (HR1), heptad repeat 2 (HR2), central helix (CH), transmembrane domain (TM), and C-terminal domain (CTD). (**B–D**) Infectivity of SARS-CoV-2 pseudoviruses. SARS-CoV-2 pseudoviruses were produced in HEK293T cells treated with 1 mM NB-DNJ, 1 mM castanospermine, or 1 μg/mL tunicamycin. The viruses were then used to infect HEK293T-ACE2 (**B**), Caco-2 (**C**), and Calu-3 (**D**) cells. Luciferase activity was analyzed as the fold change relative to that of the WT group (*n* = 3). (**E and F**) Infectivity of SARS-CoV and MERS-CoV pseudoviruses. (**G**) Infectivity of SARS-CoV-2 variants of concern (VOCs) in Caco-2 cells. Pseudoviruses were produced in HEK293T cells with or without 1 mM NB-DNJ treatment. Caco-2 cells were used as targets, and luciferase activity was analyzed as the fold change relative to that of the WT group (*n* = 3). (**H**) Replication of SARS-CoV-2 △N-GFP (MOI = 0.01) in Caco-2-N cells in the presence of N-glycosylation inhibitors (1 mM NB-DNJ, 1 mM castanospermine, or 1 μg/mL tunicamycin). GFP fluorescence was observed 24 h post-infection via fluorescence microscopy. (**I**) Quantitation of SARS-CoV-2 △N-GFP (MOI = 0.01)-infected Caco-2-N cells via flow cytometry analysis. The data represent three biological replicates. The gating of the analysis is shown in Fig. S1E. (**J and K**) Replication of authentic SARS-CoV-2. A549-ACE2 cells were infected with SARS-CoV-2 SH01 strain (MOI = 0.01), in the presence or absence of 1 mM NB-DNJ. Cells were harvested, and viral N gene mRNA levels were determined by RT-PCR (**J**). Viral S protein cleavage was analyzed by WB (**K**). (**L**) Cell-cell fusion mediated by the SARS-CoV-2 S protein. HEK293T cells were transfected with wild-type S and GFP plasmids. Twenty-four hours post-transfection, the spike-expressing cells were cocultured with HEK293T-ACE2 cells, and the GFP fluorescence was visualized via a fluorescence microscope after 8 h of coculture. (**M**) Western blot analysis of S protein cleavage and S1 subunit shedding induced by sACE2 in wild-type spike-expressing cells. sACE2 was added 4 h before cell collection. The supernatants were subjected to S1 immunoprecipitation via anti-FLAG M2 beads, with heavy and light chains serving as loading controls for supernatant IP. (**N**) Western blot analysis was performed to assess the effects of sACE2 (with or without NB-DNJ) on WT pseudovirus packaging, S protein cleavage, and S1 subunit shedding. The cells and supernatants were collected 4 h after sACE2 addition, and virions were purified from the supernatants via ultracentrifugation (*n* = 3). (**O**) Schematic diagram of the experimental design for SARS-CoV-2 infection in K18-ACE2 mouse model (*n* = 5). (**P–R**) Effect of NB-DNJ treatment on SARS-CoV-2 infection in a mouse model. K18-ACE2 mice treated with PBS or NB-DNJ were infected with BA.5.2. Infectious progeny viruses in lung tissue were determined by focus-forming units (FFUs) (**P**). Total viral N gene mRNA levels in lungs were measured by RT-PCR (**Q**). Lung tissues were analyzed by H&E staining and Immunofluorescence (**R**).

Since the emergence of SARS-CoV-2 in 2019, dozens of variants have evolved. Through sequence alignment, we found that the N-glycosylation sites on the S protein of the variants are highly conserved (Fig. 1A; Fig. S2A). To determine the impact of N-glycosylation on SARS-CoV-2 infectivity, we employed an HIV-1 lentiviral vector encoding a secreted nano-luciferase reporter and pseudotyped it with the SARS-CoV-2 spike (S) glycoprotein to assess viral infectivity (47). Three N-glycosylation inhibitors—N-butyldeoxynojirimycin (NB-DNJ), castanospermine, and tunicamycin—were used during SARS-CoV-2 production. Among these, NB-DNJ and castanospermine inhibit alpha-glucosidase, leading to impaired N-glycan synthesis (48), whereas tunicamycin blocks N-glycosylation by suppressing N-acetylglucosamine-1-phosphate transferase (GPT), thereby disrupting N-glycan synthesis and inducing ER stress (49). Progeny virions produced from inhibitor-treated cells were used to infect HEK293T-ACE2, Caco-2, and Calu-3 cell lines to measure viral infectivity. The data revealed that all three N-glycosylation inhibitors dramatically suppressed SARS-CoV-2 infectivity in the tested cell lines (Fig. 1B through D), as well as that of SARS-CoV and MERS-CoV (Fig. 1E and F). Notably, NB-DNJ exhibited consistent inhibitory effects across multiple SARS-CoV-2 variants (Fig. 1G; Fig. S1A). The NB-DNJ and castanospermine inhibitors exhibited minimal cytotoxicity within the tested concentration range, while tunicamycin exhibited approximately 25% cell mortality at the effective drug concentration (Fig. S1B through D). To validate these findings, we employed a replication-competent SARS-CoV-2 DN-GFP replicon (lacking the nucleocapsid gene) in Caco-2-N cells (stably expressing N protein) (50). The data revealed that all three inhibitors significantly reduced SARS-CoV-2 DN-GFP replication in Caco-2-N cells (Fig. 1H and I; Fig. S1E), which was consistent with the results obtained from the pseudovirus assay. Furthermore, using authentic SARS-CoV-2 (SH01 strain), we confirmed that NB-DNJ exhibited dramatic inhibitory effects in A549-ACE2 cells (Fig. 1J and K).

Syncytium formation has been demonstrated to facilitate viral replication, dissemination, and immune evasion while inducing cytopathic effects and extensive tissue damage—a phenomenon observed across numerous viral species (51–54). The SARS-CoV-2 S protein, a highly glycosylated viral fusogen that mediates membrane fusion and syncytium formation, has become a major research focus (55). To examine whether N-glycosylation contributes to S protein-mediated membrane fusion, we performed cell-cell fusion assays using a coculture system of HEK293T cells expressing the SARS-CoV-2 S protein and HEK293T-ACE2 cells, with or without the addition of NB-DNJ. Our results showed that, compared with the control group (the first row), the wild-type (D614G) S protein significantly enhanced cell-cell fusion and syncytium formation between HEK293T and HEK293T-ACE2 cells (the second row). In contrast, spike-mediated cell-cell fusion and syncytium formation were markedly reduced upon treatment with 1 mM NB-DNJ (the third row) (Fig. 1L). These data conclusively demonstrate that inhibition of N-glycosylation impairs S protein-mediated cell-cell fusion and syncytium formation.

Moreover, the cleavage site (Arg-Arg-Ala-Arg) at the S1-S2 junction represents a distinctive feature of SARS-CoV-2 that differentiates it from SARS-CoV and other known sarbecoviruses (38). The cleavage site within the S protein is cleaved by furin in the Golgi apparatus before the viral particles are released (41). Such cleavage facilitates S1 subunit shedding and exposes the S2′ cleavage site for recognition by either cell surface TMPRSS2 or endosomal cathepsins, thereby fully activating the fusion process and enabling viral cell entry. Previous studies have reported multiple N-glycosylation sites near the S1/S2 cleavage site, prompting us to investigate whether N-glycosylation influences S protein S1/S2 cleavage and S1 subunit shedding. To test this hypothesis, we expressed the S protein with both N- and C-terminal FLAG tags in HEK293T cells and induced S1 subunit shedding via soluble human ACE2 (sACE2). Immunoprecipitation assays of the collected supernatants showed that, compared with the control treatment, the 1 mM NB-DNJ treatment largely reduced the level of shed S1 subunits, and S1 shedding was dependent on sACE2 concentration in a dose-dependent manner (Fig. 1M). Analyzing the cell lysates revealed comparable expression levels of full-length spikes between the two groups, yet NB-DNJ treatment markedly decreased the number of cleavage-generated S1 subunits (Fig. 1M). We further conducted pseudovirus experiments by producing pseudoviral particles in HEK293T cells with or without NB-DNJ treatment, followed by ultracentrifugation purification. Western blot analysis revealed that NB-DNJ treatment significantly reduced the levels of S1 subunits in both viral particles and producer cells, with a slight reduction in p24 levels in the viral particles (Fig. 1N).

To verify the inhibitory effect of NB-DNJ *in vivo*, K18-hACE2 mice were infected with SARS-CoV-2 BA.5.2, with or without NB-DNJ treatment, and lung tissues were collected for analysis (Fig. 1O). FFA results showed a significant reduction in focus-forming units (FFUs) in the NB-DNJ-treated group, indicating that the number of infectious viral particles was markedly lower than that in the PBS group (Fig. 1P). RT-PCR analysis revealed significantly decreased viral RNA levels in the NB-DNJ-treated group (Fig. 1Q). HE staining showed that compared with the control group, the NB-DNJ-treated group exhibited more intact alveolar structure, remarkably reduced inflammatory cell infiltration, and milder pathological damage. IF staining further confirmed the reduced viral nucleocapsid (N) protein in the NB-DNJ group (Fig. 1R). Collectively, these findings demonstrate that NB-DNJ treatment significantly inhibits SARS-CoV-2 infection in K18-hACE2 mice, thereby decreasing virus-induced lung injury.

Together, these findings strongly suggest that N-glycosylation plays a key role in regulating SARS-CoV-2 infectivity and S protein-mediated membrane fusion by modulating S1/S2 cleavage.

### N61D and N343D glycosylation site mutations disrupt spike-mediated membrane fusion and reduce SARS-CoV-2 infectivity

**Fig 2:**
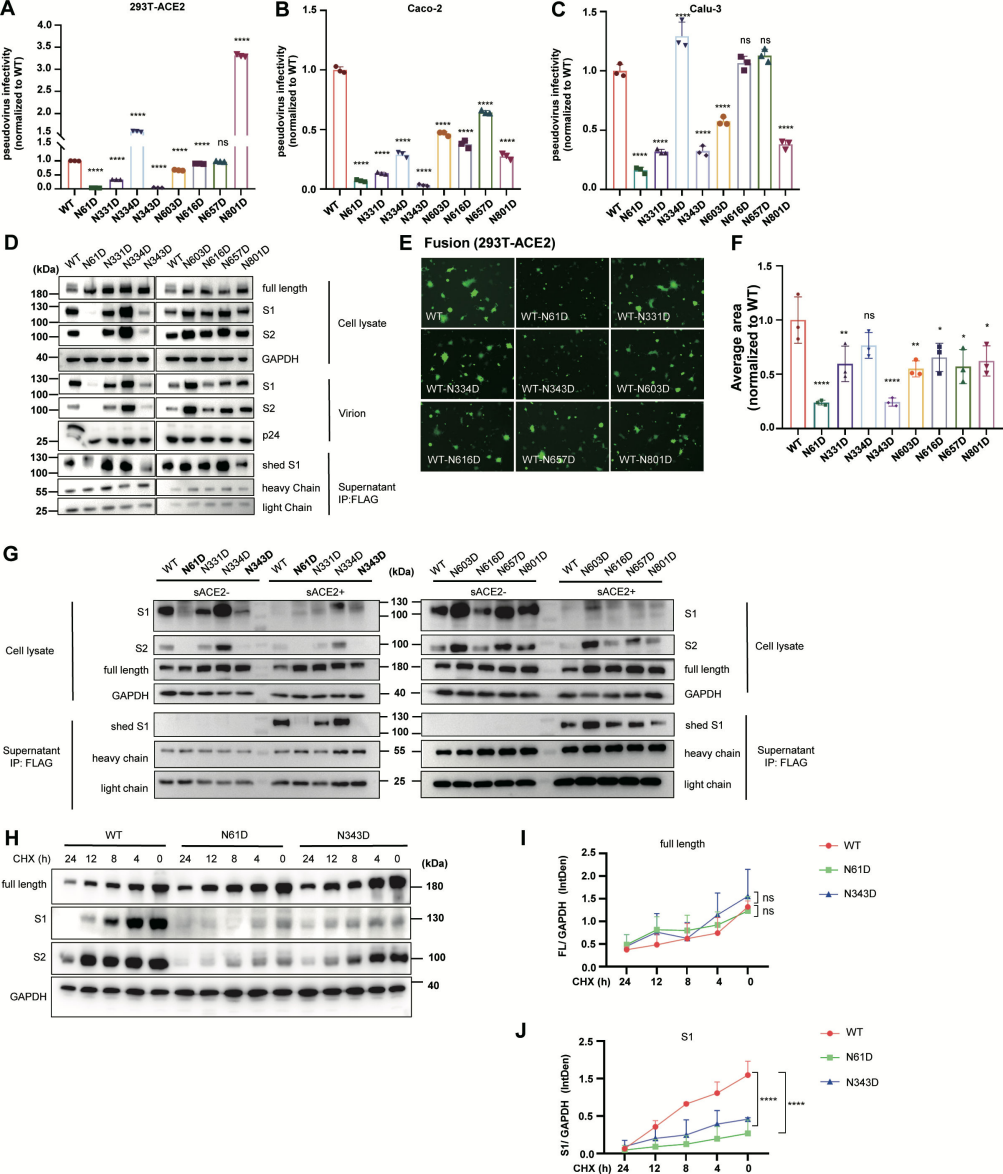
N61D and N343D glycosylation site mutations disrupt spike-mediated membrane fusion and reduce SARS-CoV-2 infectivity. (**A–C**) The infectivity of SARS-CoV-2 pseudotype viruses with S proteins harboring N-glycosylation site mutations was measured in HEK293T-ACE2 (**A**), Caco-2 (**B**), and Calu-3 (**C**) cells. (**D**) Western blot analysis was conducted to detect S proteins in viral producer cells, purified virions, and cell culture supernatant fractions. The virions were concentrated by centrifugation. After removal of virions, the S1 subunits were immunoprecipitated from supernatant with anti-FLAG agarose affinity resin. GAPDH and p24 were used as loading controls for cell lysate and virion samples, while antibody heavy/light chains served as internal controls for immunoprecipitation assays. (**E and F**) Cell-cell fusion activity mediated by S proteins harboring N-glycosylation site mutations was evaluated in HEK293T-ACE2 cells. Representative images are shown (**E**), and cell-cell fusion was quantified by measuring the fluorescence area via ImageJ software (**F**). The data are presented as relative values normalized to those of WT controls (*n* = 3). (**G**) Western blot analysis of S1/S2 cleavage of mutant spikes and S1 subunit shedding induced by sACE2. S protein-expressing cells were treated with sACE2 for 4 h, after which the cell lysate was harvested, and shed S1 was pulled down from the supernatant and analyzed by western blotting. (**H–J**) Western blot analysis of wild-type, N61D, and N343D S protein synthesis rates. The cells were treated with 20 μM cycloheximide (CHX) at the indicated time points and analyzed via western blotting (**H**). The intensities of the full-length spike and S2 subunits were quantified via ImageJ (**I and J**).

Previous studies have reported that the SARS-CoV-2 S protein has 22 N-glycosylation sites (21, 56). Our sequence alignment analysis revealed that these N-glycosylation sites are extremely highly conserved across all SARS-CoV-2 variants (Fig. S2A). To investigate the functional significance of these conserved N-glycosylation sites during viral evolution and to identify specific sites that modulate viral infectivity, we systematically generated single-point mutations at N-glycosylation sites proximal to key structural domains or crucial functional regions of the S protein, specifically the receptor-binding domain (RBD) and furin cleavage motif (Fig. 1A). By substituting asparagine with aspartic acid (N to D) to mimic glycan removal, we constructed eight mutant spikes: N61D, N331D, N334D, N343D, N603D, N616D, N657D, and N801D. Compared with the wild-type spike, all these mutant S variants disrupted the infectivity of SARS-CoV-2 pseudovirus, with the N61D and N343D mutations showing the most pronounced inhibitory effects (>99% reduction in HEK293T-ACE2 and Caco-2 cells, >70% reduction in Calu-3 cells) (Fig. 2A through C). We further analyzed the S mutants bearing indicated N-to-D mutations in viral producer cells, virions, and supernatant. The data revealed that S1/S2 cleavage of N61D and N343D was dramatically decreased in both cell lysates and virions, with comparable levels of full-length spikes in cell lysates and p24 in virion (Fig. 2D). Meanwhile, the shed S1 subunits of N61D and N343D were also decreased in cell culture supernatant (Fig. 2D). To investigate whether the reduced S1/S2 cleavage caused by N61D and N343D mutations affects the SARS-CoV-2 entry pathway, we used camostat to inhibit TMPRSS2, a protease essential for plasma membrane entry. As expected, these mutant spikes mediated cell entry through the endosomal route rather than plasma membrane route (Fig. S2H). We subsequently examined the effects of the N61D and N343D mutations on SARS-CoV-2 variants of concern (VOCs) and found that these two mutations resulted in broad-spectrum inhibition of SARS-CoV-2 VOCs in our assays (Fig. S2B and C). Subsequent cell-cell fusion assays, performed by coculturing HEK293T cells expressing mutant S proteins with HEK293T-ACE2 cells at a 1:1 ratio, revealed that the N61D and N343D mutations also significantly reduced cell-cell fusion by approximately 5-fold (Fig. 2E and F). Since spike-mediated cell-cell fusion critically depends on the binding between cell-surface spikes and the ACE2 receptor, we measured the ACE2 binding ability of these mutant spikes by flow cytometry. The results revealed significantly reduced ACE2 binding to cell-surface N61D/N343D spikes, likely due to either impaired binding affinity or decreased surface S expression (Fig. S2D and E).

We further analyzed the S1 subunit shedding of these S protein mutants induced by sACE2, which may account for the mutation-induced alterations in membrane fusion and viral infectivity. HEK293T cells expressing FLAG-tagged spikes were treated with or without sACE2, followed by immunoprecipitation with anti-FLAG beads to isolate shed S1 subunits, which were then detected via western blotting. The data revealed that the N61D and N343D mutants presented significantly impaired S1/S2 cleavage efficiency, as demonstrated by markedly reduced levels of both the S1 and S2 subunits in the cell lysates compared with those in the wild-type cells, despite comparable expression of the full-length S protein (Fig. 2G). Upon sACE2-induced S1 subunit shedding, both N61D and N343D spikes presented diminished S1 subunit release in the supernatant, with N343D exhibiting the most pronounced effect (Fig. 2G). These findings indicate that N-glycosylation at positions N61 and N343 is critical for S protein processing and maturation. Interestingly, we observed that the N334D, N603D, and N657D mutations conversely enhanced both S1/S2 cleavage and sACE2-induced S1 shedding.

To exclude the potential effects of aberrant N-glycosylation on protein synthesis that might account for our observations, we conducted the following experiments. HEK293T cells expressing wild-type, N61D, or N343D S proteins were treated with cycloheximide (CHX) at the indicated time points (24, 12, 8, 4, and 0 h prior to cell harvest) to block new protein synthesis (57), enabling analysis of S protein synthesis kinetics. Western blot data and quantitative analysis revealed equivalent full-length spike synthesis kinetics between the wild-type and N61D/N343D mutants (Fig. 2H and I), suggesting that N-glycosylation at these two sites is dispensable for S protein synthesis. In striking contrast, both the N61D and N343D exhibited significantly reduced accumulation of furin-cleaved S1 subunits compared with the wild type (Fig. 2H and J), consistent with their previous phenotype. Together, these results demonstrate that N-glycosylation at spike residues N61 and N343 is essential for SARS-CoV-2 infectivity by promoting S1/S2 cleavage, without altering S protein synthesis levels.

### Loss of N-linked glycosylation at N61/N343 abrogates spike protein membrane localization

**Fig 3:**
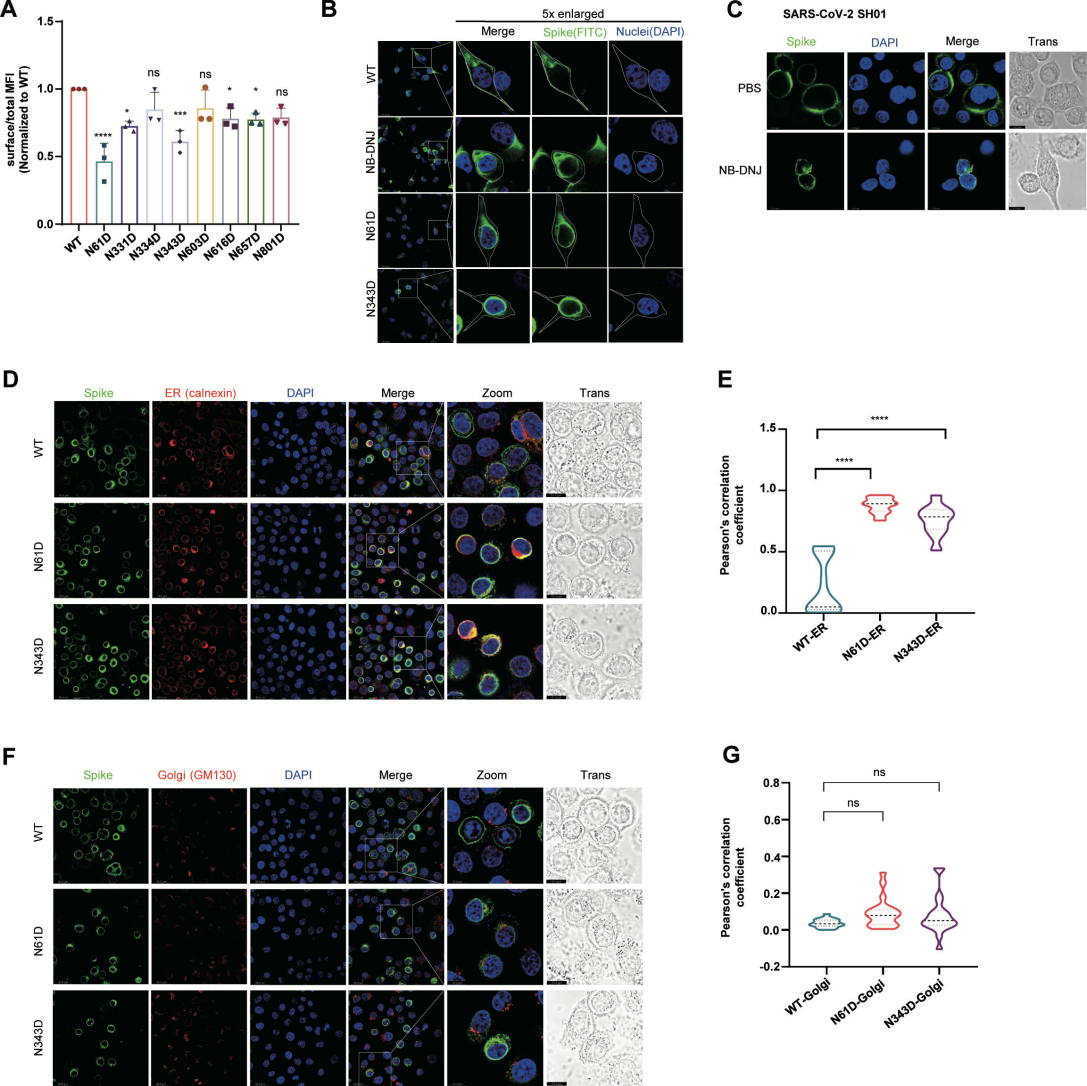
Loss of N-linked glycosylation at N61/N343 abrogates spike protein membrane localization. (**A**) Detection of S protein localization by flow cytometry through surface staining and total cellular staining (*n* = 3 biological replicates). (**B**) Representative fluorescence images of HeLa cells expressing S protein (green). The cell nuclei were stained with DAPI (blue). The data are representative of three independent experiments. (**C**) Fluorescence staining images of S protein localization in live virus-infected cells with or without NB-DNJ treatment. (**D and E**) Representative fluorescence images of the colocalization of the S protein (green) and the ER marker calnexin (red). The cell nuclei were stained with DAPI (blue) (**D**). The data are representative of three independent experiments. The colocalization of the S protein with the ER was analyzed via Pearson correlation coefficient analysis (**E**). (**F and G**) Representative fluorescence images of HeLa cells coexpressing S protein (green) and the Golgi marker GM130 (red). The cell nuclei were stained with DAPI (blue) (**F**). The data are representative of three independent experiments. The colocalization of the S protein with the ER was analyzed via Pearson correlation coefficient analysis (**G**).

To investigate how these glycosylation-deficient mutations in the S protein affect S1/S2 cleavage and subsequent plasma membrane localization, we conducted the following experiments. First, we examined the expression levels of these mutant spikes on the cell surface. HEK293T cells were transfected with the wild-type spike plasmid or those containing N-glycosylation site mutations. At 48 h post-transfection, the cells were harvested and analyzed by flow cytometry for both total S protein expression (intracellular staining) and surface S protein expression (surface staining). Compared to the other spikes, the cell surface expression of the N61D and N343D spikes was reduced when normalized to the total protein expression level (Fig. 3A; Fig. S2F).

Next, we investigated the subcellular localization of the S protein following impairment of N-glycosylation. Immunofluorescence microscopy analysis showed that the wild-type S protein exhibited characteristic plasma membrane localization. In striking contrast, either treatment with 1 mM NB-DNJ or introduction of the N61D/N343D mutation significantly attenuated the plasma membrane localization of the S protein, resulting in predominant perinuclear localization (Fig. 3B). A similar phenotype was observed during authentic virus infection: NB-DNJ treatment altered the subcellular localization of the S protein from plasma membrane to intracellular compartment (Fig. 3C). Furthermore, immunofluorescence staining of the S protein with calnexin (an ER marker) and GM130 (a Golgi marker) revealed that N61D and N343D mutant spikes were predominantly localized to the ER, with minimal plasma membrane localization and no significant Golgi apparatus localization (Fig. 3D and F). Pearson correlation coefficient analysis of spike-FITC colocalization with ER marker confirmed enhanced ER retention of N-glycosylation-deficient S proteins (Fig. 3E), whereas Golgi colocalization remained unaltered (Fig. 3G). This mislocalization suggested that impaired S1/S2 cleavage of N61D and N343D mutants is caused by their ER retention rather than by disruption of the furin cleavage site. To confirm this, *in vitro* furin cleavage assays were performed, revealing that both mutants can be cleaved by furin (Fig. S2G). These results indicate that impaired N-glycosylation disrupts S protein trafficking, leading to ER retention and consequent failure of furin-mediated S1/S2 cleavage, which affects viral assembly and infectivity.

### The COP I complex contributes to the ER retention of aberrantly N-glycosylated spike proteins

**Fig 4:**
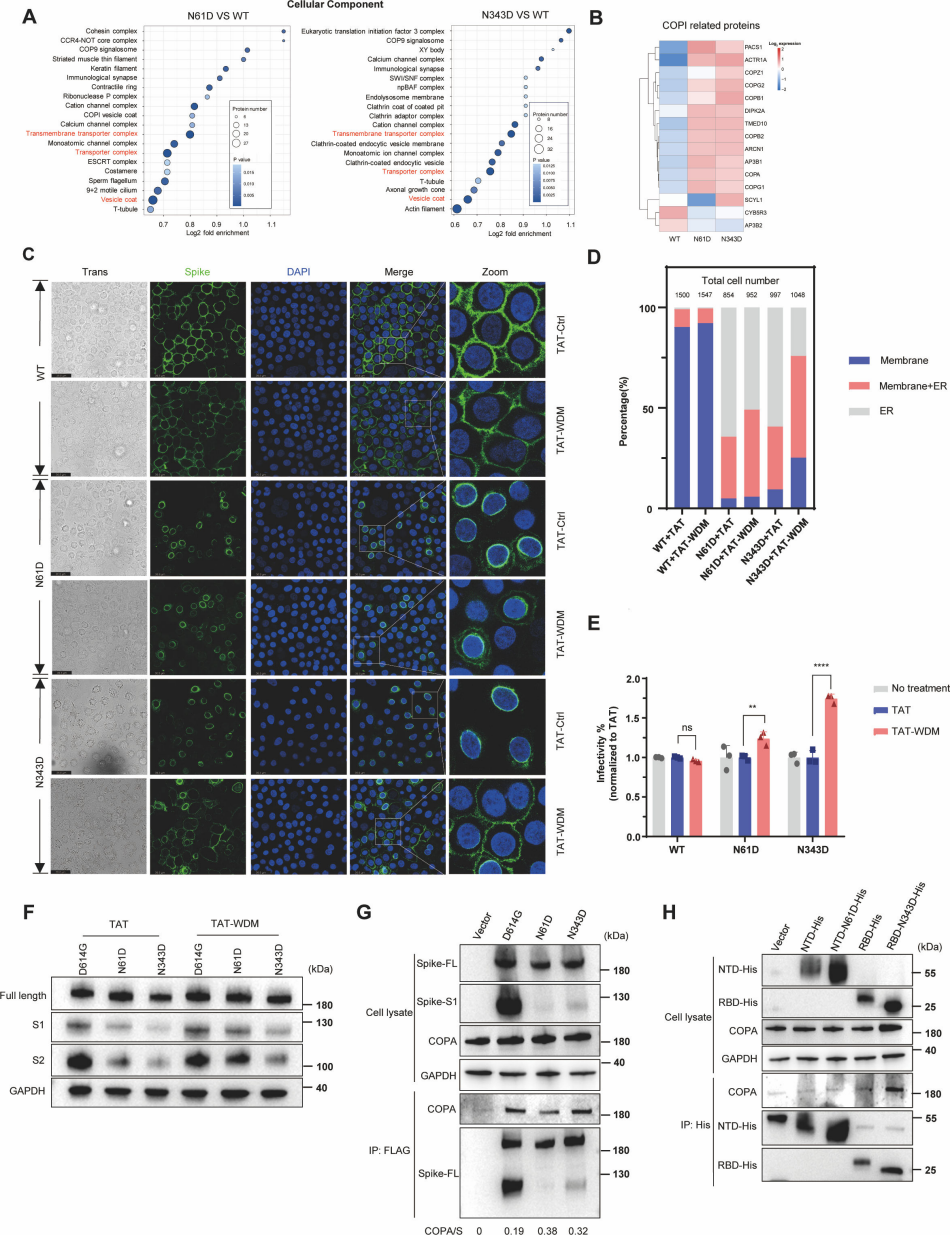
The COP I complex contributes to the ER retention of aberrantly N-glycosylated spike proteins. (**A**) Dot plot analysis of GO enrichment for differentially expressed proteins in the wild-type, N61D, and N343D groups. The horizontal axis represents the log2-transformed fold enrichment of functional categories, and the vertical axis shows GO functional descriptions. Fisher’s exact test was used to calculate the significant *P* values. (**B**) Heatmap analysis of COPI complex-related proteins identified as prominent among the differentially expressed proteins in the wild-type, N61D, and N343D groups through GO enrichment analysis. Fisher’s exact test was used to calculate the significant *P* values. (**C**) Representative fluorescence images of HeLa cells expressing S proteins (green) treated with 20 μM TAT or TAT-WDM. The cell nuclei were stained with DAPI (blue). The data are representative of three independent experiments. (**D**) Quantitative analysis of S protein membrane localization. The numerical labels on the stacked bar representing each experimental group denote the total number of cells included in the statistical analysis, ensuring the reliability of quantitative results. The blue indicates that the spike localizes to the plasma membrane, the red indicates that the spike localizes to both plasma membrane and ER, and the gray indicates that the spike localizes to ER. (**E**) Infectivity of SARS-CoV-2 wild-type, N61D, and N343D pseudoviruses in HEK293T-ACE2 cells. Viral producer cells were treated with 20 μM TAT or TAT-WDM, and the collected viruses were used to infect HEK293T-ACE2 cells, followed by luciferase activity measurement. Luciferase activity was analyzed as the fold change relative to the TAT treatment control (*n* = 3). (**F**) The S1/S2 cleavage efficiency of S protein in the absence or presence of TAT-WDM inhibitor. (**G**) Co-IP analysis of the interaction between S protein and endogenous COPA. (**H**) Co-IP analysis of the interaction between endogenous COPA and NTD (14–305) or RBD (319–541) domain of S protein.

To identify the host proteins responsible for the ER retention of the N61D/N343D S protein, we employed coimmunoprecipitation (co-IP) coupled with liquid chromatography-tandem mass spectrometry (LC-MS/MS)-based proteomics. Briefly, FLAG-tagged wild-type and mutant (N61D and N343D) S proteins were expressed in HEK293T cells, followed by immunopurification with anti-FLAG agarose affinity resin. The pulled-down proteins were analyzed via mass spectrometry. Comparative analysis of the wild-type and N61D/N343D S protein interactomes identified site-specific N-glycosylation-dependent bonding partners (Fig. S3A). Gene Ontology (GO) term analysis of cellular components revealed that the differentially expressed proteins were enriched in transport complexes, particularly vesicle coat components (Fig. 4A). COPI (coat protein I) and COPII (coat protein II), the two major types of vesicle coats that mediate protein trafficking between the ER and Golgi apparatus (58, 59), may contribute to the retention of the N61D/N343D S protein in the ER. This is especially true for COPI vesicle coat, which showed significant enrichment in the N61D versus D614G group (Fig. 4A). Heatmap analysis exhibited significant upregulation of COPI complex-associated proteins among the N61D/N343D-interacting proteins compared with the wild-type protein (D614G) (Fig. 4B), suggesting that the COPI complex may mediate retrograde transport (movement from the Golgi back to the ER) of these two N-glycosylation-deficient S mutants and contribute to their ER retention.

To test this potential mechanism, we employed the cell-penetrating peptide TAT-WDM, a previously reported suppressor of COPI-mediated protein transport (60), with the TAT peptide serving as the control (Fig. S3B). The immunofluorescence results demonstrated that TAT-WDM markedly restored the membrane localization of the N343D S protein and partially restored that of the N61D S protein while having a minimal effect on the wild-type S protein, which normally predominantly localizes to the plasma membrane (Fig. 4C and D). We further performed pseudovirus infection assays to test the efficacy of this inhibitor. Specifically, HEK293T cells were treated with or without 20 μM COPI inhibitor (TAT-WDM) during pseudovirus packaging. Wild-type or N61D/N343D mutant viruses were harvested 48 h post-transfection and used to infect HEK293T-ACE2 cells. Viral infectivity was measured 36 h post-infection. Inspiringly, TAT-WDM treatment partially rescued the infectivity of the N61D/N343D mutant viruses (Fig. 4E), which was consistent with the change in the localization of the S proteins (Fig. 4C). Nonetheless, it is worth noting that the absolute infectivity of N61D/N343D spike-pseudotyped viruses remained profoundly low, regardless of TAT-WDM treatment (Fig. S3C). Moreover, TAT-WDM treatment partially restored the S1/S2 cleavage of N61D/N343D mutant spikes (Fig. 4F). The TAT and TAT-WDM peptides showed no cytotoxicity within the tested concentration range (Fig. S3D and E).

To confirm the interaction between the COPI complex and S protein identified by IP-MS, we selected COPA (COPI Coat Complex Subunit Alpha) for further verification. Co-IP analysis showed that endogenous COPA could be pulled down by both WT spike and N61D/N343D mutant spikes, with markedly higher pulldown efficiency for the mutant spikes than for the WT spike (Fig. 4G), which was consistent with IP-MS results (Fig. 4B). As N61 is located in the NTD and N343 in the RBD domain, we questioned whether COPI interacts directly with these domains. To address this, we examined the interaction between endogenous COPA and NTD, NTD-N61D, RBD, and RBD-N343D. Data showed that RBD-N343D, but not wild-type RBD, strongly interacted with COPA, suggesting that RBD serves as a binding region for COPI and that N-glycosylation at N343 blocks this interaction (Fig. 4H).

These data demonstrate that the COPI complex contributes to ER retention of N61D and N343D glycosylation-deficient S proteins, consequently reducing viral infectivity, which can be partially restored by TAT-WDM peptide treatment.

### Blockade of the COPII-spike protein interaction impairs SARS-CoV-2 infectivity by disrupting spike trafficking

While the COPI complex facilitates retrograde transport (Golgi to ER), the COPII complex mediates anterograde ER-to-Golgi protein trafficking. Therefore, we hypothesized that specific inhibition of the COPII-spike protein interaction would prevent efficient spike trafficking to the Golgi and plasma membrane, leading to ER retention and S1/S2 inefficient cleavage, thereby mimicking the reduction in SARS-CoV-2 infectivity induced by N-glycosylation deficiency. This strategy presents a potential target for the development of antiviral drugs against SARS-CoV-2.

**Fig 5:**
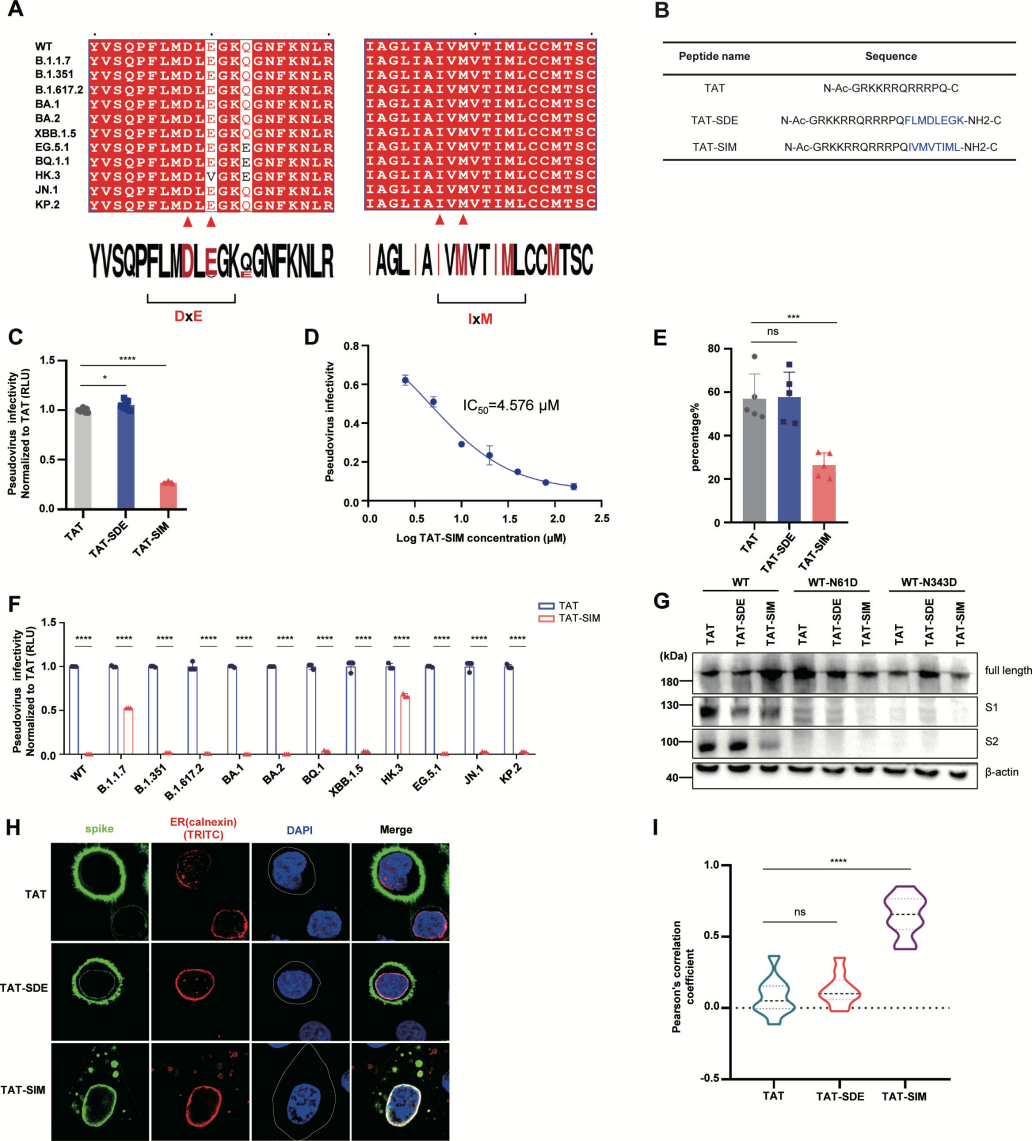
Blockade of the COPII-spike protein interaction impairs SARS-CoV-2 infectivity by disrupting spike trafficking. (**A**) Sequence conservation analysis of putative COPII binding motifs in the S protein. The alignments of the targeting sequences across the 12 SARS-CoV-2 variants are shown, with red triangles highlighting the COPII recognition motifs DxE and IxM. The sequence alignment results were obtained from the ESPript 3.0 website. (**B**) Amino acid sequences of the designed TAT-SDE and TAT-SIM cell-penetrating peptides. These two peptides each consist of a TAT cell-penetrating sequence fused to a spike-derived COPII competing binding sequence designed to interfere with the spike protein-COPII interaction. N-Ac denotes N-terminal acetylation, and NH₂-C indicates C-terminal amidation. TAT served as the control. (**C**) Infectivity of the SARS-CoV-2 pseudovirus in HEK293T-ACE2 cells. Viral producer cells were treated with 80 μM TAT, TAT-SDE, or TAT-SIM for 10 h, and the collected viruses were used to infect HEK293T-ACE2 cells, followed by luciferase activity measurement. Luciferase activity was analyzed as the fold change relative to the TAT control group (*n* = 3). (**D**) Half-maximal inhibitory concentration (IC₅₀) of TAT-SIM peptides against SARS-CoV-2. The X-axis represents the logarithm-transformed TAT-SIM concentration [log(x)], which was analyzed via nonlinear regression (curve fit) via GraphPad Prism 8. (**E**) Replication of SARS-CoV-2 △N-GFP in Caco-2 cells after treatment with TAT, TAT-SDE, or TAT-SIM. The infected cells were quantified via flow cytometry and are presented as a percentage plot. The original images are shown in S4D Fig. (**F**) Broad-spectrum inhibition of the TAT-SIM peptide against 12 SARS-CoV-2 variants. Viral producer cells were treated with 80 μM TAT or TAT-SIM, and the collected viruses were used to infect HEK293T-ACE2 cells, followed by luciferase activity measurement. Luciferase activity was analyzed as the fold change relative to the TAT group (*n* = 3). (**G**) Western blot analysis of S1/S2 cleavage in wild-type, N61D, and N343D-transfected cells. The cells were treated with 80 μM TAT, TAT-SDE, or TAT-SIM. (**H and I**) Representative fluorescence images of the colocalization of the S protein (green) and the ER marker calnexin (red). The cell nuclei were stained with DAPI (blue) (**H**). The data are representative of three independent experiments. The colocalization of the S protein with the ER was analyzed via Pearson correlation coefficient analysis (**I**).

To achieve this objective, we characterized the structural features or motifs recognized by the COPII complex and discovered two conserved motifs (DxE and IxM) in the SARS-CoV-2 S protein (61–63). Sequence alignment showed exceptional conservation of these two motifs across SARS-CoV-2 variants, particularly the IxM motif (Fig. 5A). Based on these motifs, we designed two COPII-competitive binding peptides conjugated with cell-penetrating domains to facilitate intracellular delivery and activity, naming them TAT-SDE and TAT-SIM (Fig. 5B, TAT: cell-penetrating peptide, SDE: spike DxE motif, SIM: spike IxM motif). Both peptides exhibited minimal cytotoxicity (Fig. S4A and B). To evaluate these peptides, viral producer cells were treated with TAT (control), TAT-SDE, or TAT-SIM. The collected pseudoviruses were then used to infect HEK293T-ACE2 cells for quantification of infectivity. The results indicated that TAT-SIM treatment markedly reduced SARS-CoV-2 infection (by ~3.7-fold reduction; IC_50_ = 4.576 µM), while TAT-SDE treatment exhibited comparable infectivity to that of the TAT control (Fig. 5C and D). The inhibitory effects of these peptide inhibitors were also tested in the SARS-CoV-2 ΔN-GFP replicon system, and the results exhibited a similar trend: TAT-SIM potently inhibited SARS-CoV-2 ΔN-GFP replication in Caco-2-N cells (Fig. 5E; Fig. S4D). We further tested the inhibition effect of TAT-SIM peptide across SARS-CoV-2 variants and found that TAT-SIM exhibited broad-spectrum antiviral efficacy against these variants (Fig. 5F). Mechanistic studies demonstrated that TAT-SIM treatment effectively inhibited the cleavage of the wild-type S protein S1/S2, whereas TAT-SDE had minimal effects. Both peptides had negligible effects on S1/S2 cleavage of the N61D and N343D mutants because of their low baseline cleavage efficiency (Fig. 5G). Subcellular localization assays revealed that TAT-SDE minimally affected S protein trafficking, whereas TAT-SIM significantly reduced spike plasma membrane localization and caused predominant ER localization (Fig. 5H and I). Meanwhile, TAT-SIM treatment induced the formation of spike-containing vesicular structures (potentially ER-derived vesicles), which were not observed in the other experimental groups (Fig. 5H). To determine whether TAT-SIM specifically inhibits SARS-CoV-2 S protein trafficking, we examined the cellular localization of the host cell membrane protein SERINC5 as a control. The results showed that TAT-SIM treatment did not alter the plasma membrane localization of SERINC5, indicating the specific inhibition of SARS-CoV-2 S protein transport (Fig. S4C). Collectively, these results demonstrate that the conserved IxM motif in the SARS-CoV-2 S protein is essential for COPII-mediated ER-to-Golgi trafficking, and that the engineered TAT-SIM peptide targeting this motif exhibits potent, broad-spectrum antiviral activity against SARS-CoV-2 variants.

### MOGS deficiency alters the localization of the spike protein, thereby inhibiting SARS-CoV-2 infection

It has been reported that cells from MOGS-CDG patients naturally resist infection by various viruses, but there are few reports on SARS-CoV-2 (12). Only one *in vitro* study has shown that the MOGS inhibitor has no effect on spike-mediated receptor binding and cell fusion of SARS-CoV-2 (64). Whether MOGS-CDG patients exhibit resistance to SARS-CoV-2 infection remains unclear. Given that MOGS is an essential enzyme in the protein N-glycosylation pathway, we hypothesized that MOGS deficiency-induced abnormal N-glycosylation of the S protein leads to its ER retention and consequent impairment of SARS-CoV-2 infectivity, thereby conferring viral resistance in MOGS-CDG patients.

**Fig 6:**
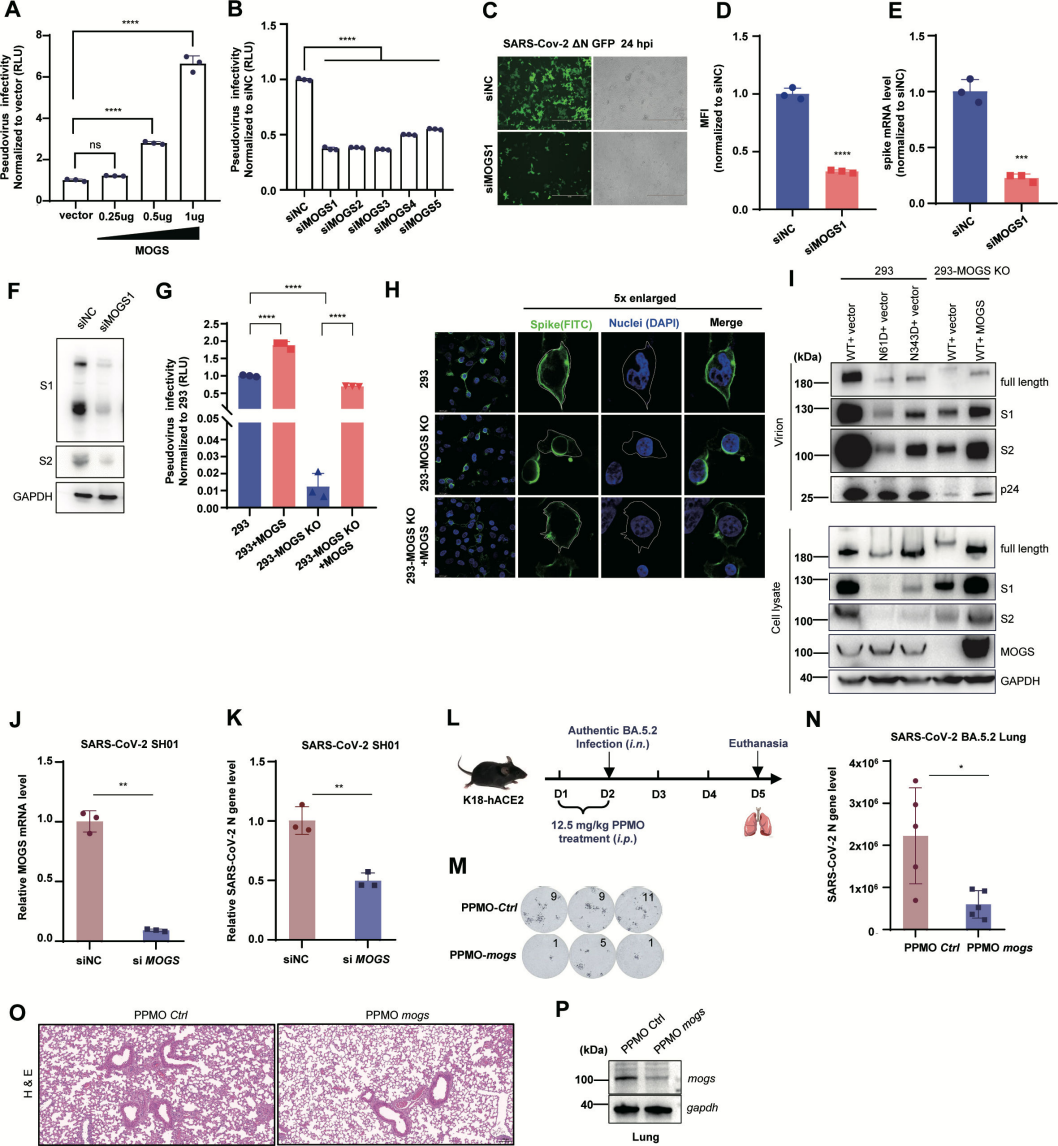
MOGS deficiency alters the localization of the spike protein, thereby inhibiting SARS-CoV-2 infection. (**A**) Overexpression of MOGS promoted SARS-CoV-2 infectivity in a dose-dependent manner. Pseudoviruses were produced in HEK293T cells transfected with different amounts of MOGS, and the collected viruses were used to infect HEK293T-ACE2 cells, followed by measurement of luciferase activity. Luciferase activity was analyzed as the fold change relative to the vector group (*n* = 3). (**B**) Knockdown of MOGS inhibited SARS-CoV-2 pseudovirus infectivity. Pseudoviruses were produced in HEK293T cells transfected with different siRNAs targeting MOGS, and the collected viruses were used to infect HEK293T-ACE2 cells for infectivity measurement. Luciferase activity was analyzed as the fold change relative to that in the siNC group (*n* = 3). (**C–F**) Knockdown of MOGS inhibited SARS-CoV-2 △N-GFP replication in Caco-2-N cells. SARS-CoV-2 △N-GFP (MOI = 0.01) was inoculated into Caco-2-N cells transiently transfected with MOGS siRNA, and fluorescence was visualized via fluorescence microscopy (**C**). The infected cells were quantified by flow cytometry (**D**) (*n* = 3). The mRNA of the viral spike gene was quantified by RT-PCR (**E**). S1/S2 cleavage was analyzed by western blotting (**F**). (**G**) Effect of MOGS knockout on SARS-CoV-2 infectivity. Pseudoviruses were produced in HEK293 cells, HEK293 cells overexpressing MOGS, HEK293-MOGS KO cells, and HEK293-MOGS KO cells with MOGS reconstitution. The collected viruses were used to infect HEK293T-ACE2 cells for infectivity measurement (*n* = 3). (**H**) Representative fluorescence images of spike (green) localization in HEK293 cells, HEK293-MOGS-KO cells, and HEK293-MOGS-KO cells with MOGS reconstitution. The cell nuclei were stained with DAPI (blue). (**I**) Western blot analysis of pseudovirus production and S1/S2 cleavage in HEK293 and HEK293-MOGS-KO cells. Virions were purified from supernatants by ultracentrifugation. (**J and K**) Replication of authentic SARS-CoV-2 (SH01, MOI = 0.01) in A549-ACE2 cells with siNC or siMOGS treatment. MOGS and SARS-CoV-2 N gene mRNA levels were analyzed by RT-PCR. (**L**) Schematic diagram of the experimental design for SARS-CoV-2 infection in K18-ACE2 mice with PPMO treatment. (**M–P**) Effect of *mogs* knockdown on SARS-CoV-2 infection in K18-ACE2 mice. K18-ACE2 mice treated with PPMO-ctrl or PPMO-mogs were infected with BA.5.2. Infectious progeny viruses in lung tissue were determined by focus-forming units (FFUs) (**M**). Total viral N gene mRNA levels in lungs were measured by RT-PCR (**N**). Representative images of H&E staining for lung tissues (**O**). Western blot analysis of *mogs* knockdown efficiency following PPMO treatment.

To test this hypothesis and determine the role of MOGS in SARS-CoV-2 infection, we conducted the following experiments. First, MOGS was overexpressed in viral producer cells, and the pseudoviruses were harvested and used to infect HEK293T-ACE2 cells for infectivity measurement. MOGS overexpression increased pseudovirus infectivity in a dose-dependent manner (Fig. 6A; Fig. S5A).

Second, five different siRNAs were used to knock down endogenous MOGS during pseudovirus production. This knockdown significantly reduced viral infectivity (Fig. 6B), and the knockdown efficiency is shown in Fig. S5B. Furthermore, the overall effect of MOGS knockdown was confirmed using a replication-deficient SARS-CoV-2 ΔN-GFP virus in Caco-2-N cells, which strongly (~3.2-fold) suppressed viral replication (Fig. 6C and D). This knockdown also decreased spike expression and impaired S1/S2 cleavage (Fig. 6E and F).

Third, a CRISPR-Cas9-mediated MOGS knockout HEK293 cell line was generated for further investigation (Fig. S5C). Compared with parental HEK293 cells, MOGS knockout led to a dramatic (98%) reduction in SARS-CoV-2 pseudovirus infectivity. Importantly, supplementation with exogenous MOGS rescued the infectivity to near the wild-type level (Fig. 6G). Localization analysis of the S protein revealed that in MOGS-deficient HEK293 cells, the S protein accumulated in perinuclear regions (likely the ER), whereas MOGS supplementation restored its plasma membrane localization (Fig. 6H). Moreover, S1/S2 cleavage was markedly decreased in both MOGS-KO cells and progeny virions, resulting in a similar phenotype to that of the N61D and N343D mutant S proteins. As expected, the reintroduction of MOGS into MOGS-deficient HEK293 cells rescued this impaired S1/S2 cleavage (Fig. 6I). Notably, knockout of MOGS reduced p24 levels and caused a distinct shift in the molecular weight of the S protein, likely resulting from complete loss of function of α-Glc3Man9GlcNAc2 oligosaccharide glucosidase (α-glucosidase I), indicating that MOGS deficiency not only affects S protein processing but also suppresses overall pseudovirus production (Fig. 6I).

Furthermore, we validated the role of MOGS during authentic SARS-CoV-2 infection both *in vitro* and *in vivo*. siRNA-mediated knockdown of MOGS significantly reduced SARS-CoV-2 (SH01) infection in A549-ACE2 cells (Fig. 6J and K). In K18-ACE2 mice, PPMO-mediated knockdown of *mogs* dramatically decreased BA.5.2 infection (Fig. 6L through P). Compared with the control group, the PPMO-mogs treatment group exhibited fewer infectious viral particles and lower total viral RNA levels in the lungs (Fig. 6M and N). Moreover, HE staining of lung tissues revealed milder pathological lesion in the PPMO-mogs treatment group (Fig. 6O). The knockdown efficiency is shown in Fig. 6P. Together, these results demonstrate that MOGS plays a key role for SARS-CoV-2 during viral infection.

These findings suggest that MOGS, as the first enzyme in the N-linked oligosaccharide modification processing pathway, plays a crucial role in SARS-CoV-2 spike N-glycosylation and maturation, thereby influencing the infectivity of SARS-CoV-2. Furthermore, these results offer a mechanistic basis for the viral resistance phenotype characteristic of MOGS-CDG patients.

## DISCUSSION

N-glycosylation of viral proteins is a key regulatory step in viral infection (65–67). The SARS-CoV-2 S protein, which is known to be highly N-glycosylated, has been shown to mediate critical infection processes, including receptor binding and immune evasion (26, 68, 69). Several studies have shown that glycosylation regulates intracellular trafficking of SARS-CoV-2 spike protein, although the underlying mechanism remains unelucidated (70, 71). Here, we identify a distinct regulatory mechanism, whereby site-specific N-glycosylation precisely modulates the intracellular trafficking of the S protein.

Our work revealed that N-glycosylation contributes to the maturation of SARS-CoV-2 S protein, with inhibition causing (i) disrupted membrane localization of S protein and (ii) impaired S1/S2 cleavage, consequently diminishing progeny virion infectivity (Fig. 1 and 3). Through systematic mutagenesis, we identified N61 and N343 as essential N-glycosylation sites contributing to these processes (Fig. 2 and 3). While previous studies linked N343 glycosylation to conformational changes in the RBD motif and receptor binding during viral entry (not observed in the Delta variant) (46), our study demonstrated an evolutionarily conserved role for both N61 and N343 in intracellular S protein trafficking across SARS-CoV-2 variants. The broad-spectrum inhibitory effects of glycosylation defects at these conserved sites suggest their indispensable role in SARS-CoV-2 replication. Additionally, beyond the universal role of N-glycosylation at the N61 and N343 residues identified in this study for all SARS-CoV-2 variants, previous studies have reported nonuniversal effects whereby the loss of potential glycans at spike residues N717 and N801 specifically inhibits infectivity for Omicron but not for D614G or Delta variants (72), indicating a complex and delicate regulatory mechanism of spike N-glycosylation during viral infection.

Mechanistically, we demonstrated that the loss of glycosylation at N61 or N343 triggers COPI-dependent ER retention, thereby disrupting the normal S protein trafficking route and leading to inefficient S1/S2 cleavage (Fig. 4). Notably, while the COPI sorting inhibitor TAT-WDM was previously shown to enhance SARS-CoV-2 spike membrane localization without mechanistic details (60), our results not only confirmed these findings but also further established the functional linkage between N61/N343 glycosylation and COPI-mediated spike trafficking. Specifically, properly N-glycosylated spikes normally transit from the ER through the Golgi to the plasma membrane, whereas N-glycosylation-deficient spikes undergo COPI-dependent retrograde transport from the Golgi back to the ER, impairing S1/S2 processing and plasma membrane localization. However, whether the ER retention of the S protein upon loss of N61/N343 glycosylation results from direct binding of these sites to the transport machinery or from indirect regulatory effects remains unclear. Notably, the partial rescue of N61D/N343D mutant infectivity by the COPI peptide inhibitor TAT-WDM (particularly for N343D), with incomplete restoration of plasma membrane localization and S1/S2 cleavage compared to that of the wild type, suggests either limited inhibitor efficacy or additional underlying mechanisms, such as receptor binding (46).

Current studies have shown that inhibitors targeting the maturation of S protein, such as furin protease inhibitors, effectively suppress SARS-CoV-2 infection. However, long-term blockade of furin, which is required for normal development, might be associated with unwanted toxic effects (73). Therefore, inhibitors targeting the maturation process of S protein beyond the furin cleavage should be considered. On the basis of the opposing activities of COPI and COPII in protein transport, we developed a novel COPII-binding peptide inhibitor, named TAT-SIM, that competitively disrupts the spike protein-COPII interaction to mimic the COPI-mediated ER retention of N61D/N343D spikes (Fig. 5). Interestingly, the TAT-SIM peptide induced potent ER retention and inefficient S1/S2 cleavage, exhibiting broad-spectrum activity against SARS-CoV-2 variants. In addition to the TAT-SDE and TAT-SIM described in this paper, we also designed a peptide inhibitor targeting DEDDSE (1257-1262), which is the COPII binding site for ER export at the cytoplasmic tail of the S protein (43). However, the efficacy was limited and was not shown in the manuscript. Targeting viral intracellular trafficking has been a novel strategy in recent years. Targeting AAK1 and GAK, which regulate viral intracellular trafficking by controlling clathrin-associated AP complexes, has shown promise as a broad-spectrum antiviral approach (74). Peptide targeting the ARF4 protein is also an ideal target, which blocks the transport of newly formed viruses to the Golgi complex and instead diverts them to the lysosomal degradation pathway, ultimately inhibiting viral release (75). These antiviral strategies, as well as our TAT-SIM peptide, share the core idea of “targeting viral protein transport.” Although further optimization is needed for TAT-SIM to reduce cytotoxicity and enhance inhibitory efficacy, this approach highlights the therapeutic potential of specifically disrupting spike trafficking.

Furthermore, our findings reveal the positive effects of MOGS on SARS-CoV-2 infection and provide mechanistic insights into the viral resistance observed in MOGS-CDG patients (Fig. 6). However, a previous study reported that the MOGS inhibitor Miglustat has limited effect on SARS-CoV-2, with evidence demonstrating the effects on only S protein-mediated receptor binding and cell fusion (64). While our research primarily demonstrated the impact on viral proteins, we cannot exclude the possibility that MOGS-CDG patients may also achieve antiviral effects through the modulation of host proteins. It should be noted that MOGS is essential for survival, and MOGS-CDG patients typically have severely shortened lifespans, with only 24 cases described (11). Therefore, we were unable to obtain patient samples for *in vivo* experiments, which is a limitation of this work.

Since generating mutations in authentic virus was challenging, all experiments on the N61D and N343D mutations in this study were performed using pseudoviruses. Thus, the mechanistic interpretation of the N61D and N343D mutations for authentic SARS-CoV-2 remains incomplete, which represents a limitation of our work. Moreover, HIV-based pseudoviruses assemble and bud at the plasma membrane relying on spike trafficking, whereas authentic viruses assemble in the ERGIC, representing another limitation of this study. Notably, we used authentic SARS-CoV-2 in our inhibitor treatment assays, MOGS knockdown experiments, and *in vivo* studies, all of which support our conclusion that N-glycosylation is crucial for S protein trafficking and essential for SARS-CoV-2 infection.

**Fig 7:**
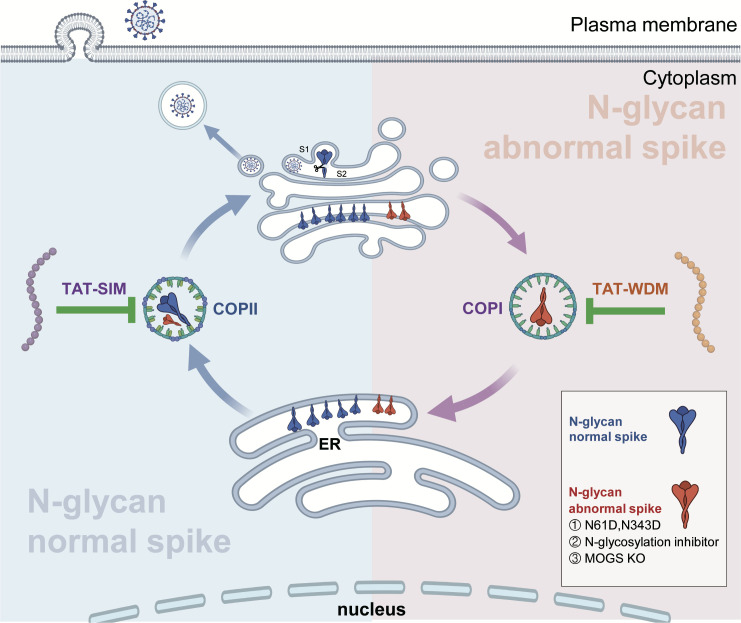
Schematic diagram of the possible role of N-glycosylation in regulating spike maturation and SARS-CoV-2 replication. (Left) Normal N-glycosylated spike proteins are transported from the ER to the Golgi through the COPII vesicles, followed by furin-mediated S1/S2 cleavage. The mature spike proteins are packaged into viral particles, which are then released from host cells and thus form infectious progeny viruses. This ER-Golgi transport process can be specifically and effectively inhibited by TAT-SIM, a COPII competitive binding inhibitor designed in this study. (Right) On the other hand, spike proteins that fail to undergo normal N-glycosylation (including mutations at key N-glycosylation sites, inhibitor treatment, and defects in critical enzymes involved in the N-glycosylation process) are retrogradely transported to the ER through COPI vesicles. This results in the retention of spike proteins in the ER, prevents them from undergoing subsequent maturation steps, and leads to failure in effectively forming infectious viral particles. TAT-WDM, a COPI inhibitor, can inhibit the ER retention of these N-glycan abnormal spikes and restore the viral infectivity.

In summary, this study not only reveals a pioneering mechanism by which N-glycosylation of the S protein regulates SARS-CoV-2 replication but also develops a broad-spectrum anti-SARS-CoV-2 inhibitor (Fig. 7), thereby providing unique insights and a theoretical foundation for developing broad-spectrum therapeutic strategies against SARS-CoV-2.

## MATERIALS AND METHODS

### Cell lines

All the cell lines were maintained at 37°C under 5% CO_2_ and were routinely tested for mycoplasma contamination. The Huh-7 (RRID: CVCL-0336), HEK293T (Cat# CL0169; RRID:CVCL_1926), Caco-2 (Cat# PWE-HU027; RRID: CVCL-0025), and Calu-3 (Cat# PWE-HU052; RRID: CVCL-0609) cell lines were purchased from Meilun. The HeLa cell line (Cat# FH0314; RRID: CVCL-0030) was purchased from Fuheng. The HEK293 and HEK293-MOGS-KO cell lines were gifts from Professor Xiaodong Gao (Jiangnan University). The Caco-2-N cell line was obtained from Professor Yu Chen (Wuhan University). The HEK293T-hACE2 and A549-hACE2 cell lines were established in our lab. All cells were grown in Dulbecco’s Modified Eagle’s Medium supplemented with 10% fetal bovine serum and 1% penicillin-streptomycin.

### Microbe strains

*E. coli* DH5α was purchased from YEASEN.

The SARS-CoV-2-△N-GFP virus (modified from the Wuhan-Hu-1 strain) was provided by Prof. Yu Chen (Wuhan University).

The authentic SARS-CoV-2 SH01 (nCoV-SH01) and BA.5.2 strains were used in this study. All viruses were provided by the BSL-3 laboratory of Shanghai Medical College (Fudan University) and were originally isolated from patients in Shanghai.

### Plasmids

FLAG-tagged expression plasmids for the SARS-CoV-2 variant S proteins were cloned and inserted into the pcDNA3.1 expression vector via standard cloning techniques, and plasmids harboring site-specific N-glycosylation mutation were generated via inverse PCR. All the mutants were verified via Sanger sequencing (GENEWIZ, Suzhou, China), and the primers used for the mutations were as follows:

N61D-Fwd: 5′-TTCAGCGACGTGACATGGTTCCACGCCAT-3′

N61D-Rev: 5′-TGTCACGTCGCTGAAGAACGGCAGGAAGAGG-3′

N331D-Fwd: 5′-TTTCCAGACATTACCAACCTCTGCCCCTT-3′

N331D-Rev: 5′-GGTAATGTCTGGAAATCGCACGATGCTCTC-3′

N334D-Fwd: 5′-ATTACCGACCTCTGCCCCTTCGGCGAGGTGTTTA-3′

N334D-Rev: 5′-GCAGAGGTCGGTAATGTTTGGAAATCGCACGATGCTCTC-3′

N343D-Fwd: 5′-GTGTTTGACGCCACAAGATTTGCCTCCGTT-3′

N343D-Rev: 5′-TGTGGCGTCAAACACCTCGCCGAAGGGG-3′

N603D-Fwd: 5′-GGGACCGATACCAGCAACCAGGTGGCTGTG-3′

N603D-Rev: 5′-GCTGGTACTGGTCCCGGGGGTGATCACAGAAAC-3′

N616D-Fwd: 5′-GGCGTTGACTGCACCGAGGTTCCTGTGGCCAT-3′

N616D-Rev: 5′-GGTGCAGTCAACGCCTTGGTACAGCACAGCCA-3′

N657D-Fwd: 5′-CACGTGGATAACAGTTACGAGTGCGACATCCCT-3′

N657D-Rev: 5′-ACTGTTATCCACGTGCTCGGCGCCGATCAGACATCCCG-3′

N801D-Fwd: 5′-GGCTTTGACTTTAGCCAGATTCTCCCTGATCCTTCT-3′

N801D-Rev: 5′-GGCTAAAGTCAAAGCCGCCGAAGTCCTTGATAGGTGG-3′.

The constructs used to produce the lentiviral pseudotypes included the HIV-1-NL4.3-inSecNluc vector, which was originally obtained from Shan-Lu Liu’s laboratory at Ohio State University (Columbus, Ohio, USA) (47).

The lentiviral vector pCDH-CMV-MCS-puro was provided by Prof. Jianer Long (Fudan University) to generate the HEK293T-hACE2 cell line. Lentivirus particles were produced by transfecting HEK293T cells with the transfer plasmid pCDH-CMV-ACE2-puro, packaging plasmid psPAX2, and envelope plasmid pMD2.G. The resulting lentivirus was then used to transduce target cells.

### Antibodies and chemicals

The antibodies used for western blot analysis were as follows: anti-FLAG (Proteintech, Cat# 20543-1-AP), anti-HA (Proteintech, Cat# 51064-2-AP), anti-SARS-CoV-2 spike S1 (Sino Biological, Cat# 40591-T62), anti-SARS-CoV-2 spike S2 (Sino Biological, Cat# 40590-MM05), anti-SARS-CoV/SARS-CoV-2 nucleocapsid (Sino Biological, Cat# 40143-R001), anti-His(Beyotime, Cat# AF2870), anti-COPA (Proteintech, Cat# 83089-1-RR), anti-mouse GAPDH (Proteintech, Cat# 60004-1-Ig), anti-GCS1 (Proteintech, Cat# 17859-1-AP), anti-HIV-1 p24 (Proteintech, Cat# 11695), anti-mouse IgG (Sigma-Aldrich, Cat# A5278), and anti-rabbit IgG (Sigma-Aldrich, Cat# A9169). The antibodies used for immunofluorescence and flow cytometry were as follows: anti-calnexin (Proteintech, Cat# 10427-2-AP), anti-GM130 (Proteintech, Cat# 11308-1-AP), anti-Lamin B1 (Proteintech, Cat# 12987-1-AP), anti-mouse-FITC (Sigma-Aldrich, Cat# F0257), anti-rabbit-FITC (Sigma-Aldrich, Cat# F9887), anti-human-FITC (Sigma-Aldrich, Cat# F9512), anti-mouse-TRITC (Thermo Fisher Scientific, Cat# A16165), and anti-rabbit-TRITC (Proteintech, Cat# SA00007-2).

The peptides TAT (N-acetylation-GRKKRRQRRRPQ-C), TAT-WDM (N-acetylation-GRKKRRQRRRPQAKEKSD-C), TAT-SDE (N-acetylation-GRKKRRQRRRPQFLMDLEGK-NH2-C), and TAT-SIM (N-acetylation-GRKKRRQRRRPQIVMVTIML-NH2) were obtained from Synpeptide Company (Nanjing, China) with greater than 95% purity.

Reagents, including Miglustat (NB-DNJ) (Cat# HY-17020), Castanospermine (Cat# HY-N2022), tunicamycin (Cat# HY-A0098), furin (Cat# HY-P78747), and Camostat mesylate (Cat# HY-13512), were purchased from MedChemExpress (MCE). Cycloheximide (Cat# S7418) was purchased from Selleck. Puromycin (Cat# 60209ES10), DAPI Stain Solution (Cat# 40728ES03), and permeabilization wash buffer (Cat# 40403ES64) were obtained from Yeasen. TRIzol reagent (Cat# 15596026CN), Lipofectamine 2000 (Cat# 11668019), and Lipofectamine RNAiMAX (Cat# 13778150) were purchased from Invitrogen. Polyethylenimine (PEI, Cat. No. 24765-1) was purchased from Polysciences. Anti-FLAG M2 affinity gel (Cat# A2220) was purchased from Millipore. Protein G resin (Cat# L00209-50) was purchased from GenScript. IgG Elution Buffer (Cat# 21004) was purchased from Thermo Scientific. The Nano-Glo Luciferase Assay System (Cat# N1120) was purchased from Promega. The SuperKine Maximum Sensitivity Cell Counting Kit-8 (Cat# BMU106-CN) was purchased from Abbkine.

Soluble ACE2 (sACE2) was produced using the plasmid pFUSE-hIgG1-Fc2-ACE2 (a gift from Prof. Qiao Wang, Fudan University). In brief, the plasmid was transfected into Expi293 cells via a PEI transfection protocol. The supernatant was harvested to purify sACE2 via protein G affinity chromatography.

### Virus production and infection

The SARS-CoV-2-△N-GFP virus was produced in Caco-2-N cells. In detail, SARS-CoV-2-△N-GFP virus (MOI = 0.01) was inoculated into a 12-well plate containing 80–90% confluent Caco-2-N cells. The virions were collected at 48 h post-infection, filtered through a 0.45 µm filter, aliquoted, and stored at −80°C. The TCID_50_ of the virus was calculated using the Reed and Muench method (50).

The pseudotyped virus was produced as previously described (76). Briefly, HEK293T cells were transfected with HIV-1-NL4.3-inSecNluc and various S protein constructs at a 2:1 ratio using PEI. The supernatants were harvested after 48 h and 72 h post-transfection, pooled, filtered through a 0.45 µm filter, aliquoted, and stored at −80°C. The pseudovirus was used to infect the target cells for 6 h, after which the medium was replaced with fresh medium. Luciferase activity was measured at 48 h post-infection using TECAN Spark microplate reader to determine viral infectivity.

The viral particles used for western blotting were concentrated via ultracentrifugation. Filtered viruses were layered on top of a 25% sucrose gradient and centrifuged at 100,000 × *g* for 90 min at 4°C. The pellet was resuspended in PBS for subsequent analysis.

### Immunofluorescence confocal microscopy

HeLa cells were cultured in 12-well glass-bottom plates until they reached 30–40% confluency. The S protein was transfected into HeLa cells via a Lipofectamine 2000 transfection protocol. At 24 h post-transfection, the cells were washed with PBS and fixed with 4% paraformaldehyde at room temperature for 10 min. Following three PBS washes, the cells were permeabilized with 0.1% Triton X-100 for 15 min and blocked with 5% bovine serum albumin (BSA) for 1 h at room temperature. After overnight incubation with the primary antibody at 4°C, the cells were incubated with the secondary antibody for 1 h at room temperature. Nuclei were stained with DAPI. Confocal fluorescence images were captured via a Leica TCS SP8 fluorescence microscope (Leica Application Suite X v.3.7.5.24914). Images were acquired using 405, 488, and 561 nm lasers.

### Immunoprecipitation-mass spectrometry (IP-MS) analysis

For immunoprecipitation-mass spectrometry mass spectrometry (IP-MS), HEK293T cells were seeded in 10 cm dishes and transfected with 15 μg (per dish) of WT, N61D, or N343D expression plasmids. At 36 h post-transfection, the cells were collected and lysed in 1 mL of prechilled RIPA lysis buffer (50 mM Tris-HCl, 150 mM NaCl, 1 mM EDTA, 1% NP-40, 0.1% SDS and complete protease inhibitor cocktail) for 30 min on ice. The cell lysates were centrifuged at 12,000 × *g* for 10 min at 4°C, and the supernatants were incubated with 40 μL of anti-FLAG M2 beads for 8 h at 4°C. The beads were washed three times with prechilled PBS, and the precipitated proteins were eluted by boiling in 1× SDS loading buffer and analyzed by western blotting.

After verifying protein expression and purification, the samples were subjected to label-free quantitative mass spectrometry. For digestion, the protein mixture was reduced with 5 mM dithiothreitol for 30 min at 56°C and then alkylated with 11 mM iodoacetamide for 15 min at room temperature in the dark. The protein sample was then diluted by adding 200 mM TEAB to a urea concentration of less than 2 M. Finally, trypsin was added at a 1:50 trypsin-to-protein mass ratio for the first overnight digestion and at a 1:100 trypsin-to-protein mass ratio for a second 4 h digestion. Finally, the peptides were desalted on a Strata X SPE column. The tryptic peptides were dissolved in solvent A and directly loaded onto a custom-made reversed-phase analytical column (25 cm in length, 100 μm in diameter). The mobile phase consisted of solvent A (0.1% formic acid in 2% acetonitrile/water) and solvent B (0.1% formic acid in acetonitrile). Peptides were separated with the following gradient: 0–9 min, B: 6–24%; 9–11 min, B: 24–35%; 11–13 min, B: 35–80%; and 13–15 min, B: 80%, with a constant flow rate of 500 nL/min on a NanoElute UHPLC system (Bruker Daltonics). The peptides were subjected to a capillary source, followed by mass spectrometry on a timsTOF Pro 2 mass spectrometer. The electrospray voltage applied was 1.75 kV. The precursors and fragments were analyzed with the TOF detector. The timsTOF Pro was operated in data-independent parallel accumulation–serial fragmentation (DIA-PASEF) mode. The full MS scan was set as 300–1500 (MS/MS scan range), and 20 PASEF (MS/MS mode) MS/MS scans were acquired per cycle. The MS/MS scan range was set to 400–850, and the isolation window was set to 7 m/z. The DIA data were processed using the DIA-NN search engine (v. 1.8). Tandem mass spectra were searched against Homo sapiens (9606) SP 20231220. fasta (20,429 entries) concatenated with a reverse decoy database. Trypsin/P was specified as a cleavage enzyme, allowing up to one missing cleavage. Excision of N-term Met and carbamidomethyl on Cys was specified as fixed modifications. The FDR was adjusted to <1%.

Fisher’s exact test was used to analyze the significance of functional enrichment of differentially expressed proteins (using the identified protein as the background). Functional terms with a fold enrichment >1.5 and a *P* value < 0.05 were considered significant. Cluster analysis based on protein functional enrichment of differentially expressed proteins (or proteins with different fold changes) was used to investigate their potential associations and differences in specific functions (GO analysis). First, the functional enrichment results for all protein groups were collected, and then functions that were significantly enriched (*P* value < 0.05) in at least one protein group were filtered out. The filtered *P* value data matrix was then subjected to a log10 transformation, followed by one-sided clustering analysis via hierarchical clustering (Euclidean distance, average linkage clustering) on the transformed data set. The clustering relationships were visualized using a heatmap generated by the heatmap function in the R package ComplexHeatmap.

### Western blotting

For western blotting, equal amounts of cell lysates were resolved via SDS-PAGE and transferred to a hybridization nitrocellulose membrane (Millipore, Cat# HATF00010). The membrane was blocked with 5% nonfat milk and incubated with appropriate primary and secondary antibodies. GAPDH and β-actin were used as controls. Three independent experiments were performed for each analysis. All band intensities were evaluated using a high-signal ECL Western Blotting Substrate (Tanon, Cat# 180-5001) and normalized to those of GAPDH. The signal intensity of the western blot bands was quantified by Image J.

### Flow cytometry

For cell surface staining of spikes, equivalent numbers of cells were collected. The cells were washed once with cold PBS containing 2% FBS (PBS/2% FBS) and then fixed in 4% paraformaldehyde for 10 min at room temperature. Following three washes with PBS/2% FBS, the cells were incubated with primary antibodies in PBS/2% FBS for 1 h on ice. After three additional washes with cold PBS/2% FBS, the cells were incubated with secondary antibodies for 1 h on ice. The cells were then washed three times with PBS/2% FBS and resuspended in cold PBS/2% FBS for flow cytometry analysis using a BD LSR-II analyzer (BD Biosciences). The data were analyzed using FlowJo (version 10.8.1).

For total S protein staining, cells were permeabilized with permeabilization wash buffer (Yeasen, Cat# 40403ES64) on ice for 15 min after fixation. Antibody staining was subsequently performed using the same protocol as for surface staining.

### Knockdown of MOGS with siRNAs

HEK293T cells were plated into six-well plates, followed by transfection with 100 nM siRNAs against *MOGS* or siRNA control using LipoRNAiMax transfection reagent. Cells were collected for RT-qPCR at 36 h after siRNA transfection. The sequences of the *MOGS* siRNAs are listed below:

H7841-si*MOGS*-1: 5′-GAUGGUAAAGAGUCGCCUA (dT)(dT)-3′

5′-UAGGCGACUCUUUACCAUC (dT)(dT)-3′

H7841-si*MOGS*-2: 5′-GGCAGUUCUUGAUACAGCA (dT)(dT)-3′

5′-UGCUGUAUCAAGAACUGCC (dT)(dT)-3′

H7841-si*MOGS*-3: 5′-GCAGUAUGUAGAUGCUCUU (dT)(dT)-3′

5′-AAGAGCAUCUACAUACUGC (dT)(dT)-3′

H7841-si*MOGS*-4: 5′-GUGACUUCCGCUUUACACU (dT)(dT)-3′

5′-AGUGUAAAGCGGAAGUCAC (dT)(dT)-3′

H7841-si*MOGS*-5: 5′-GCCCACUGCCACUAUCUUA (dT)(dT)-3′

5′-UAAGAUAGUGGCAGUGGGC (dT)(dT)-3′

### RNA extraction and RT-qPCR

Total RNA was extracted using TRIzol reagent (Invitrogen, Cat# 15596018). Reverse transcription was performed using 1 μg of purified total RNA as a template with the Hifair II 1st Strand cDNA Synthesis Kit (Yeasen, Cat# 11121ES60). cDNA samples were then used as templates for qPCR with Hieff qPCR SYBR Green Master Mix (Yeasen, Cat# 11120ES03). Relative mRNA expression levels of target genes were normalized to those of GAPDH and quantified via the 2^(−ΔΔCt) method. All the experiments were repeated at least three times. All primers used for RT-qPCR are listed below: qGAPDH-Fwd: 5′-GGAAGGTGAAGGTCGGAGTCAACGG-3′ qGAPDH-Rev: 5′-CTGTTGTCATACTTCTCATGGTTCAC-3′ qMOGS-Fwd: 5′-TCCGCTTTACACTTTTGCCAC-3′ qMOGS-Rev: 5′-GTCCAGAAGACATTGTAGCTGCC-3′ qSARS-CoV-2-N-Fwd: 5′-GGGGAACTTCTCCTGCTAGAAT-3′ qSARS-CoV-2-N-Rev: 5′-CAGACATTTTGCTCTCAAGCTG-3′ qSARS-CoV-2-S-Fwd: 5′-TCAACTCAGGACTTGTTCTTAC-3′ qSARS-CoV-2-S-Rev: 5′-TGGTAGGACAGGGTTATCAAAC-3′

### *In vivo* infection experiments

Human ACE2 overexpression transgenic mice (K18-hACE2) were obtained from Prof. Deyin Guo (Guangzhou National Laboratory). Male mice aged 10–11 weeks were randomly divided into different groups, receiving NB-DNJ treatment or PPMOs-mediated *mogs* knockdown, respectively, with control groups set up. After anesthesia, mice were intranasally inoculated with 5,000 PFU of SARS-CoV-2 BA.5.2 strain. At the end of the experiment, mice were euthanized, and lung tissues were collected for subsequent experiments including RT-PCR, HE staining, immunofluorescence (IF), and fluorescent focus-forming assay.

PPMOs used for *mogs* knockdown were purchased from Gene Tools. Specifically, the *mogs*-targeting PPMO (PPMO-*mogs*) was designed with the sequence GTCGCCTCTCGCCCCAGGCCATG, while the non-targeting control PPMO (PPMO-Ctrl) had the sequence CCTCTTACCTCAGTTACAATTTATA. K18-hACE2 mice were intraperitoneally injected with 12.5 mg/kg of PPMOs (dissolved in PBS) for 2 consecutive days.

All animal experiments were performed in strict compliance with the guidelines and have been approved by the Laboratory Animal Ethics Committee of the School of Basic Medical Sciences at Fudan University (approved number: 20230301-098).

### Fluorescent focus-forming assay (FFA)

Lung homogenates were prepared using a FastPrep24 system (MP Biomedicals). Tissue debris was removed by centrifugation, and virus titers were quantified by FFA. In detail, Vero-E6 cells (1 × 10⁴ cells/well) were seeded in 96-well plates for 18 h to form confluent monolayers. Ten-fold serially diluted viruses were incubated with cells (37°C, 1 h), overlaid with 2% carboxymethylcellulose (2% FBS), and cultured for 48 h. Cells were fixed (4% polyformaldehyde), permeabilized (1% saponin and 0.2% Triton X-100), and incubated with rabbit anti-SARS-CoV-2 nucleocapsid antibody (Sino Biological; 1:2,000) followed by an HRP-conjugated secondary antibody. Viral foci were visualized with TrueBlue substrate and counted via Immunospot analyzer (Cellular Technology Limited).

### CCK8 assay

To evaluate the cytotoxicity of the peptides and compounds, HEK293T cells were treated with different concentrations of cycloheximide, NB-DNJ, casanospermine, tunicamycin, or peptides for 24 h and then incubated with CCK8 reagent (Abbkine, Cat# BMU106-CN) for 1 h at 37°C. The absorbance of the CCK-8 formazan product was measured at 450 nm via a microplate reader to evaluate cell viability.

### Statistical analysis

The data were analyzed as the means with standard deviations (SDs). All the experiments were performed with at least three independent replications. Statistical analyses were performed using GraphPad Prism 8 (La Jolla, CA, USA) as follows: a *t*-test was used for single comparisons between two groups, and one-way ANOVA with Bonferroni post hoc correction was used for multiple comparisons among multiple groups. *P* values of less than 0.05 were considered statistically significant and are indicated by asterisks. *P < 0.05; **P < 0.01; ***P < 0.001; ****P < 0.0001; ns, not significant.

## Data Availability

The original data can be provided upon request to the corresponding author, Cong Zeng (congzeng@fudan.edu.cn).

## SUPPLEMENTAL MATERIAL

Supplemental figures jvi.00270-26-s0001.pdf Figures S1 to S5.

ASM does not own the copyrights to Supplemental Material that may be linked to, or accessed through, an article. The authors have granted ASM a non-exclusive, world-wide license to publish the Supplemental Material files. Please contact the corresponding author directly for reuse.
