# Supplementary material for: Disruption of spike protein N-glycosylation induces its endoplasmic reticulum retention and attenuates SARS-CoV-2 infectivity
Source: J Virol. 2026 Mar 30;100(4):e00270-26. doi: 10.1128/jvi.00270-26 (PMC13098204; doi:10.1128/jvi.00270-26)
Supplement: Supplemental figures — Figures S1 to S5. [file jvi.00270-26-s0001.pdf]

Figure S1

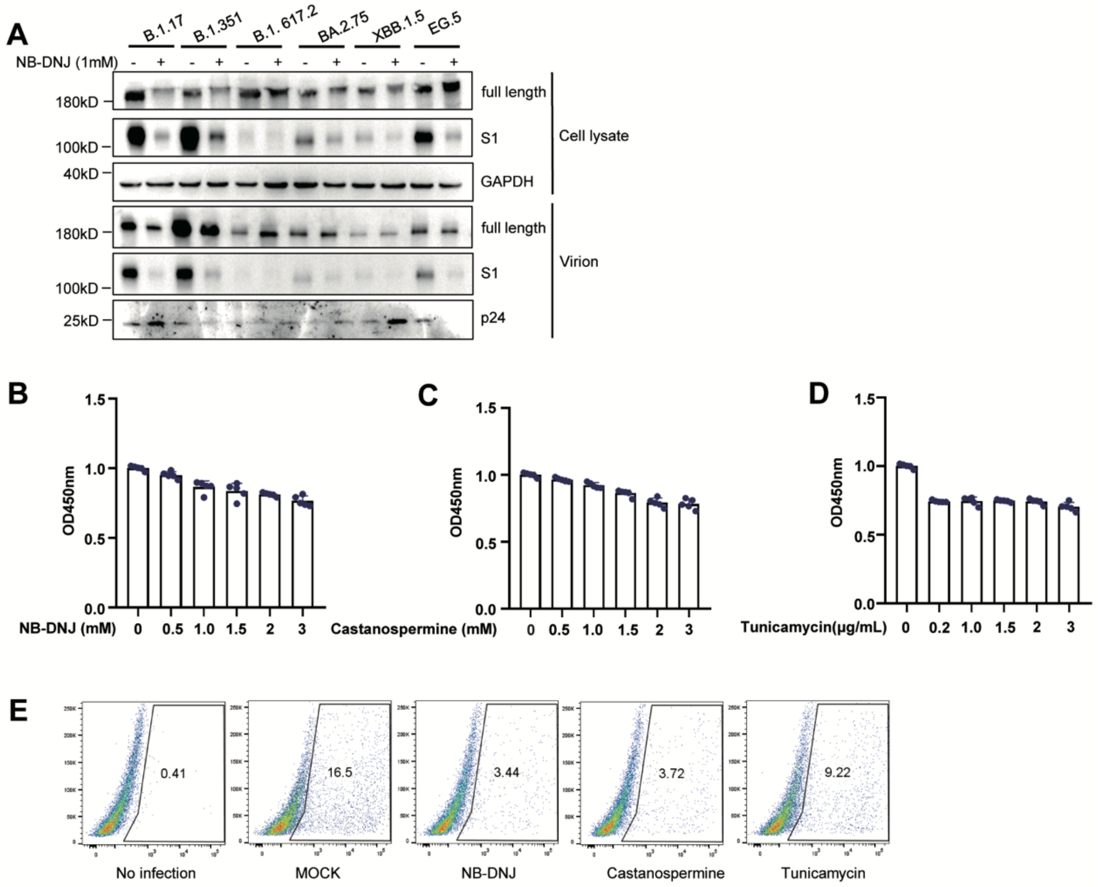

Fig S1. N-glycosylation inhibitors reduce the cleavage efficiency of the spike protein in different SARS-CoV-2 VOCs.

(A) Effects of NB-DNJ on SARS-CoV-2 VOC pseudovirus production and S1/S2 cleavage.

(B-D) Effects of N-glycosylation inhibitors on cell proliferation were assessed via a CCK8 assay. HEK293T cells were treated with increasing concentrations of N-glycosylation inhibitors for 24 h. Cell viability was measured via the CCK8 assay and normalized to that of the vehicle control group (0 μM). The data represent three biological replicates.

(E) The gating strategy for flow cytometry in Fig 1I.

Figure S2

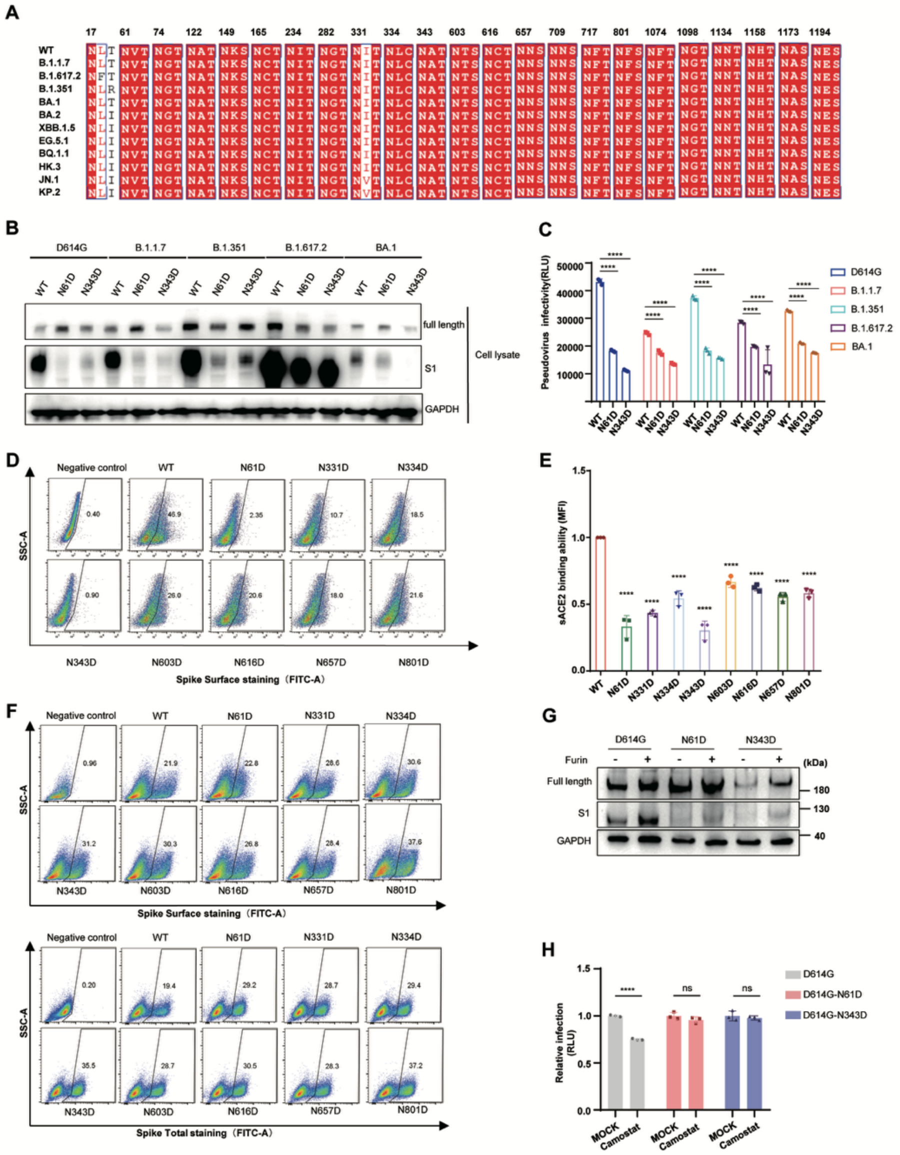

Fig S2. Conserved N61 and N343 residues are essential for S protein function and SARS-CoV-2 infection.

(A) Sequence alignment of N-glycosylation sites across different SARS-CoV-2 variants.

(B) Effects of the N61D and N343D mutations on S1/S2 cleavage in SARS-CoV-2 VOCs.

(C) Effects of the N61D and N343D mutations on SARS-CoV-2 VOCs infectivity.

(D-E) Binding ability between sACE2 and SARS-CoV-2 S mutations. The data represent three independent experiments.

(F) The gating strategy for flow cytometry in Fig 3A.

(G) In vitro furin-mediated cleavage assay of the S protein. Flag-tagged S protein was immunoprecipitated (IP) from cell lysates using anti-Flag antibody. The immunoprecipitated S protein was then subjected to in vitro cleavage with 0.5 mg/mL furin enzyme at 37 °C for 6 h. The cleavage efficiency was subsequently detected by Western blotting (WB).

(H) Verification of the cell entry pathways for N61D and N343D mutant pseudoviruses. Target cells were pretreated with Camostat (10 μM) before viral infection. Viral infectivity

was detected by chemiluminescence assay.

Figure S3

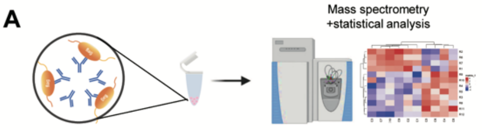

B

Peptide name	Sequence	Target
TAT	N-Ac-GRKKRRQRRRPQ-C	N/A
TAT-WDM	N-Ac-GRKKRRQRRRPQAKEKSD-C	COPI

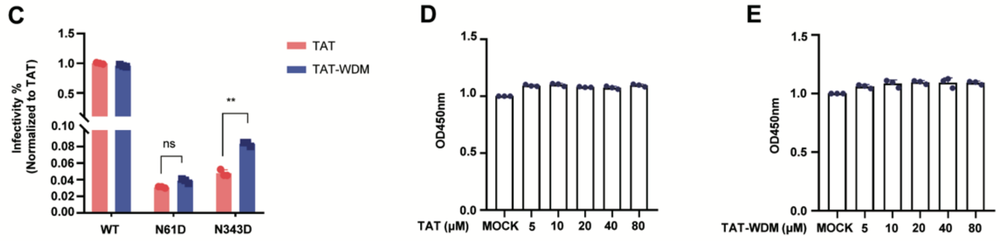

Fig S3. The inhibitor targeting COPI rescued the N61D and N343D pseudovirus infectivity.

(A) Flowchart of immunoprecipitation-mass spectrometry (IP-MS). The cell lysate was incubated with anti-FLAG beads, followed by mass spectrometry analysis and statistical evaluation.

(B) Detailed information on the TAT and TAT-WDM cell-penetrating peptides. TAT-WDM is composed of a TAT cell-penetrating sequence and a COPI-specific sequence designed for specific targeting of COPI. Nc denotes N-terminal acetylation. TAT served as the control group.

(C) Infectivity of SARS-CoV-2 WT, N61D, and N343D upon TAT-WDM treatment.

(D-E) Effects of TAT and TAT-WDM on cell proliferation were assessed via a CCK8 assay. HEK293T cells were treated with increasing concentrations of TAT and TAT-WDM for 12 h. Cell viability was measured via a CCK8 assay and normalized to that of the vehicle control group (0 mM). The data are presented as the means ± SDs (n=3 independent experiments).

Figure S4

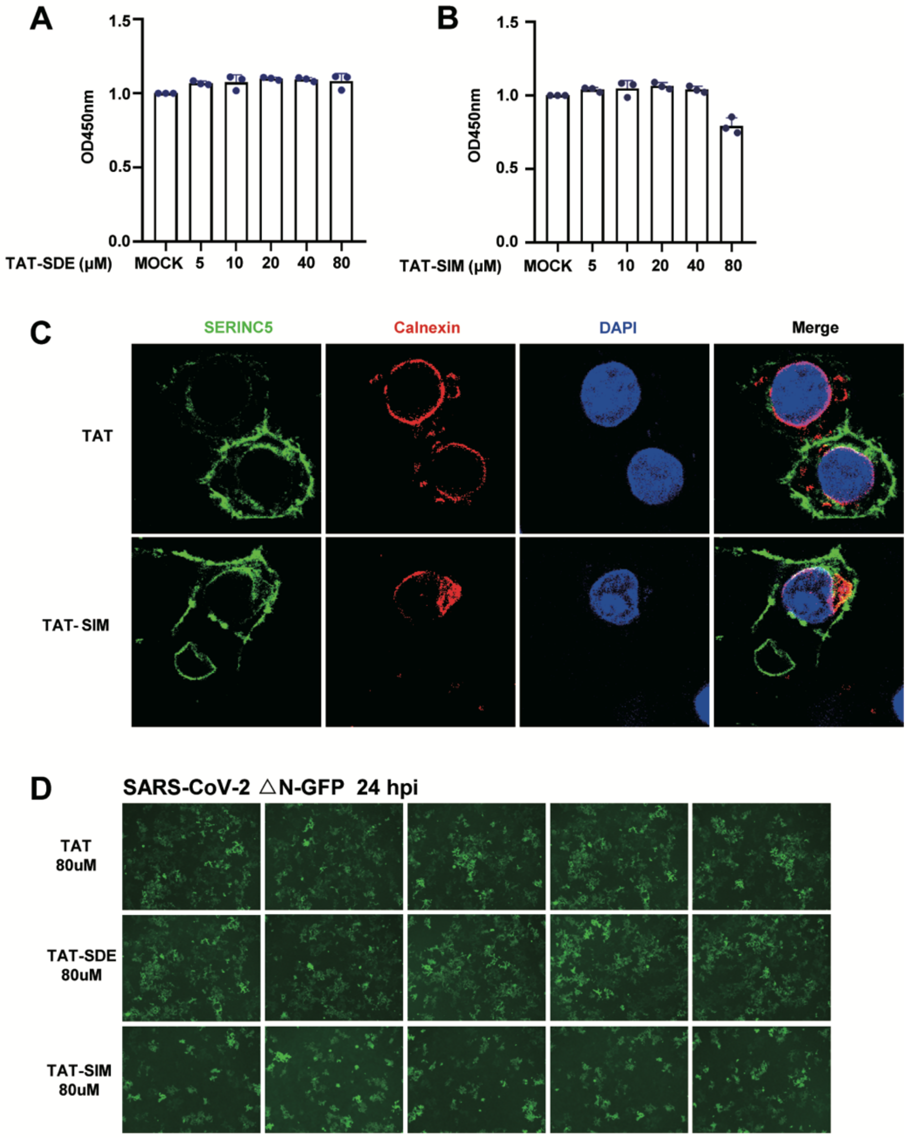

Fig S4. The peptide TAT-SIM inhibit the infection of the SARS-CoV-2-ΔN-GFP replicon.

(A-B) Effects of TAT-SDE and TAT-SIM on cell proliferation were assessed via a CCK8 assay. HEK293T cells were treated with increasing concentrations of TAT-SDE and TAT-SIM for 12 hours. Cell viability was measured via a CCK8 assay and normalized to that of the vehicle control group (0 mM). The data are presented as the means ± SDs (n = 3 independent experiments).

(C) Effect of TAT-SIM on the plasma membrane localization of SERINC5.

(D) The immunofluorescence images supporting the statistical graphs in Fig 5E.

Figure S5

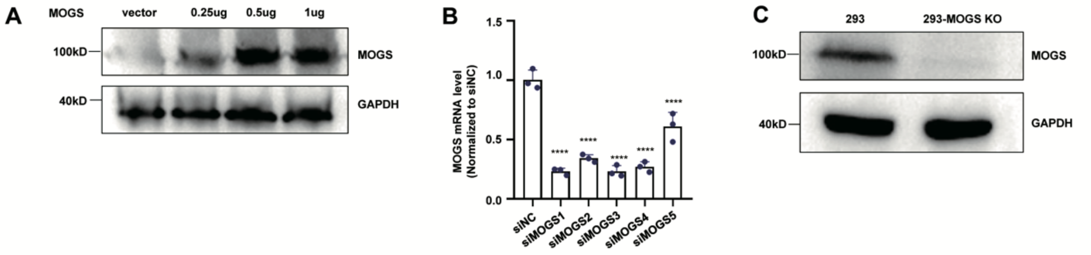

Fig S5. The expression efficiency of MOGS in different experiments.

(A) Western blot analysis of MOGS expression levels shown in Fig 6A.

(B) Quantification of MOGS mRNA levels via real-time qPCR to assess siRNA-mediated MOGS knockdown efficiency.

(C) Western blot analysis of MOGS expression levels in HEK293 and HEK293-MOGS KO cells.
